# Biomechanical analysis of the maxillary sinus floor membrane during internal sinus floor elevation with implants at different angles of the maxillary sinus angles

**DOI:** 10.1186/s40729-024-00530-5

**Published:** 2024-03-12

**Authors:** Yinxin Deng, Ruihong Ma, Yilin He, Shujia Yu, Shiyu Cao, Kang Gao, Yiping Dou, Pan Ma

**Affiliations:** 1 Dental Implant Center, Beijing Stomatological Hospital, School of Stomatology, Capital Medical University, 4 Tiantan Xili, Beijing, Dongcheng District 100050 China; 2 Department of Stomatology, Beijing Hospital of Integrated Traditional Chinese and Western Medicine, No. 3 Yongding Road East Street, Beijing, HaiDian District 100039 China

**Keywords:** Internal sinus elevation, Finite element analysis, Sinus floor membrane, Maxillary sinus angle (anatomy)

## Abstract

**Objective:**

This study analyzed and compared the biomechanical properties of maxillary sinus floor mucosa with implants at three different maxillary sinus angles during a modified internal sinus floor elevation procedure.

**Methods:**

3D reconstruction of the implant, maxillary sinus bone, and membrane were performed. The maxillary sinus model was set at three different angles. Two internal maxillary sinus elevation models were established, and finite element analysis was used to simulate the modified maxillary sinus elevation process. The implant was elevated to 10 mm at three maxillary sinus angles when the maxillary sinus floor membrane was separated by 0 and 4 mm. The stress of the maxillary sinus floor membrane was analyzed and compared.

**Results:**

When the maxillary sinus floor membrane was separated by 0 mm and elevated to 10 mm, the peak stress values of the implant on the maxillary sinus floor membrane at three different angles were as follows: maxillary sinus I: 5.14–78.32 MPa; maxillary sinus II: 2.81–73.89 MPa; and maxillary sinus III: 2.82–51.87 MPa. When the maxillary sinus floor membrane was separated by 4 mm and elevated to 10 mm, the corresponding values were as follows: maxillary sinus I: 0.50–7.25 MPa; maxillary sinus II: 0.81–16.55 MPa; and maxillary sinus III: 0.49–22.74 MPa.

**Conclusion:**

The risk of sinus floor membrane rupture is greatly reduced after adequate dissection of the maxillary sinus floor membrane when performing modified internal sinus elevation in a narrow maxillary sinus. In a wide maxillary sinus, the risk of rupture or perforation of the wider maxillary sinus floor is reduced, regardless of whether traditional or modified internal sinus elevation is performed at the same height.

**Supplementary Information:**

The online version contains supplementary material available at 10.1186/s40729-024-00530-5.

## Introduction

There is little alveolar bone available in the posterior maxillary region because of the presence of the maxillary sinus. The sparseness of bone in this region contributes to a relatively high rate of implant failure. Maxillary sinus elevation [1–3] is necessary to address this problem and can involve either more typical external sinus elevation [4] (lateral window maxillary sinus elevation via an opening in the anterior wall of the maxillary sinus) or less invasive internal sinus elevation [5, 6] (transcrestal maxillary sinus elevation via the alveolar crest). External maxillary sinus elevation [7] involves stripping the membrane of the maxillary sinus floor under direct vision with high elevation. However, this procedure involves many regions and may cause additional trauma. The surgical procedure is complex and is associated with several postoperative side effects and a lengthy recovery period. In contrast, internal maxillary sinus elevation [8, 9] involves the use of a specialized osteotome to elevate the membrane of the maxillary sinus floor. It has the advantage of being less invasive, with a shorter operation time and fewer postoperative side effects. Many factors, including the residual alveolar bone height (RBH), maxillary sinus anatomy, and surgeon expertise, should be assessed when deciding on a preoperative surgical approach. Although there is still debate over this issue, the RBH has been identified as the main factor to be considered [10, 11]. The current belief is that an internal maxillary sinus elevation procedure is relatively safe and reliable when the RBH is ≥ 4 mm. However, due to the highly sensitive nature of the technique, the application of internal maxillary sinus elevation is limited [12] when the RBH is < 4 mm.

According to some researchers, internal maxillary sinus elevation is efficient and practicable [13–15] when the RBH < 4 mm. In our clinical practice, we have also performed a modified internal sinus elevation procedure to facilitate internal sinus elevation even when the RBH < 4 mm. The modified internal sinus elevation procedure combines external maxillary sinus elevation with sinus mucoperiosteal stripping and internal sinus elevation through the top of the alveolar ridge. The membrane stripping procedure is performed on the maxillary sinus floor at the top of the alveolar ridge using a stripping instrument. This reduces the tension on the sinus mucoperiosteum, permitting greater elevation and simultaneous implant placement. We have successfully implemented the procedure in several cases. A retrospective clinical study of this modified procedure was performed, and the results [16] have been successfully published in the Journal of Clinical Implant Dentistry and Related Research. However, in our subsequent use of the modified internal sinus elevation procedure, we have found that the difference in the angle of the maxillary sinus is an equally important factor.

Perforation of the maxillary sinus floor membrane is the most common complication [17–19] of maxillary sinus floor elevation, regardless of the type of surgery [20, 21] performed or modifications [22–24] made. Our retrospective analysis of clinical cases revealed that the structural makeup of the maxillary sinus (such as the membrane thickness, width, angularity, and septal condition) is an important but frequently disregarded aspect. The morphological classification of the maxillary floor wall and the rate of perforation in maxillary sinus elevation have been noted in many studies [25–28]. Based on cone-beam computed tomography (CBCT) [29, 30] imaging data of the maxillary sinus, other scholars have studied and categorized the maxillary sinus, primarily based on two aspects: the maxillary sinus angle [31] and width [32]. Several scholars [25] have noted that the risk of membrane perforation [27] during maxillary sinus elevation is relatively increased in maxillary sinuses with small angles after combining imaging data and clinical cases. Others have shown that the width [33] of the maxillary sinus determines the distance [34] the sinus membrane can be stripped from the buccal wall to the palatal wall; the greater this distance is, the more difficult the stripping. Therefore, sufficient elevation of the membrane at the bottom of the sinus is difficult when the maxillary sinus is wider, resulting in a limited elevation height. Additionally, when the maxillary sinus is wide [35], it is more challenging for the implant material to directly contact the buccal and palatal walls of the maxillary sinus. Furthermore, the sinus floor’s membrane also collapses downward due to excessive local tension after surgery, leading to resorption of the implant material. In essence, the width of the maxillary sinus and the proportion of new bone production are inversely correlated.

The morphology of the maxillary sinus is also directly related to the difficulty of performing procedures to elevate the maxillary sinus floor, the success rate, and the incidence of complications such as membrane perforation of the maxillary sinus floor. Preoperative imaging is essential for analyzing the morphology [36, 37] of the maxillary sinus and selecting the best surgical technique. When we perform the procedure of stripping the membrane at the floor of the sinus in clinical practice, we also find that the ease of stripping the sinus floor membrane and the height to which it can be elevated vary at different angles. Additionally, osteogenesis in the maxillary sinus differs among different angles after the modified internal maxillary sinus elevation procedure. We have found that in some patients with a flat maxillary sinus floor, after modified sinus elevation, although we successfully stripped the membrane of the sinus floor and placed the implant with bone grafting material, the postoperative osteogenesis was less than optimal. However, studies on whether the sinus floor membrane or elevation height is affected differently when implants are lifted at different maxillary sinus angles have yet to be reported.

Based on the analysis described above, a three-dimensional (3D) finite element model of the implant, bone and membrane in the maxillary sinus region was established during this experiment to dynamically simulate the separation and lifting of the maxillary sinus floor membrane and study the stress and strain of the maxillary sinus floor membrane when the implant is placed in the maxillary sinus model at different angles of internal sinus elevation. While this study focused on the stresses of the maxillary sinus floor membrane at different sinus angles, some of the 3D model images and experimental data were taken from a previous study by our team [16]. Rationality was achieved in vivo but not in vitro, utilizing a technique that is not invasive. The biomechanical studies will also offer a theoretical and practical foundation for the therapeutic application of modified internal sinus elevation.

## Materials and methods

### Experimental equipment

- Hardware: NewTom VG CBCT (Italy): voltage, 110 kV; current, 3.6 mA; reconstructed layer thickness, 0.125 mm.
- Modeling software: Mimics 21.0 software (Materialise, Belgium).
- Geomagic Studio2014 software (Raindrop, USA).
- HyperMesh 14.0 software (Altair, USA).
- Finite element analysis software: MSC Patran 2012 preprocessing software (NASA, USA).
- MSC Nastran 2012 postprocessing software (NASA, USA).

### 3D finite element modeling

#### 3D model reconstruction of the implant

**Fig. 1:**
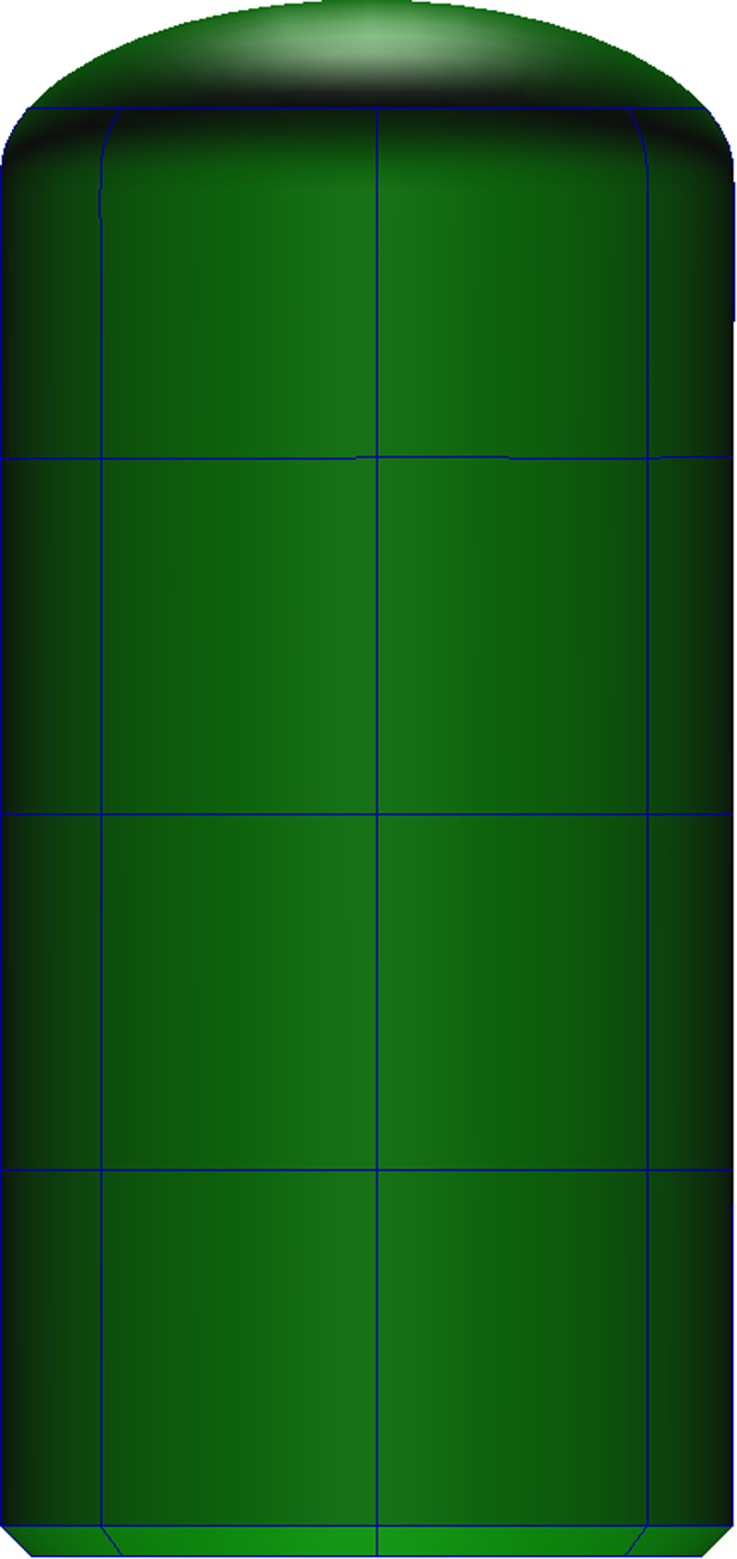
Reversed implant geometry model

The ITI Straumann bone-level cylindrical implant, which is frequently used in clinical practice [38] as a prototype, was used to establish an implant model, as shown in Fig. 1, to study the different impacts of the implant tip on the membrane of the maxillary sinus floor during implant placement at various maxillary sinus angles during maxillary sinus elevation. The implant’s bottom end had a diameter of 4.8 mm, and its overall height was approximately 10.2 mm. The implant’s apical surface was rounded and had a diameter of 4.45 mm.

### 3D model reconstruction of the maxillary sinus bone and membrane

First, 3D model reconstruction was carried out using CBCT data in DICOM format.

**Fig. 2:**
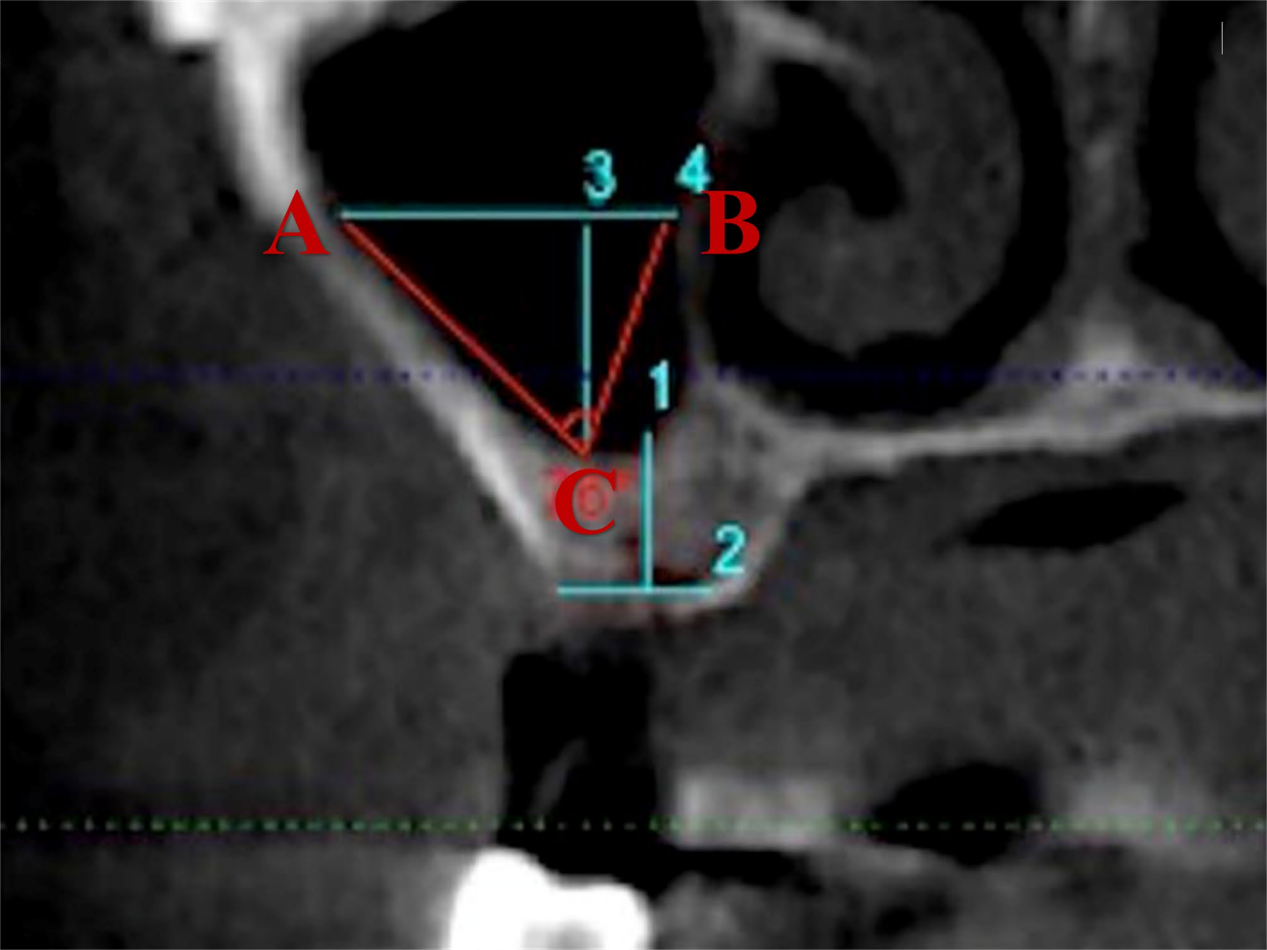
The investigated anatomical parameters [27] measured on coronal preoperative CBCT. Maxillary sinus width (L_AB_): Horizontal distance between the buccal and palatal walls of the maxillary sinus at 10 mm above the lowest point of the maxillary sinus floor at the intended implantation site. Maxillary sinus angle (∠ ACB): Angle between a horizontal line drawn 10 mm above the lowest point of the maxillary sinus floor at the intended implantation site and the buccal wall bone plate and palatal wall bone plate of the maxillary sinus

**Fig. 3:**
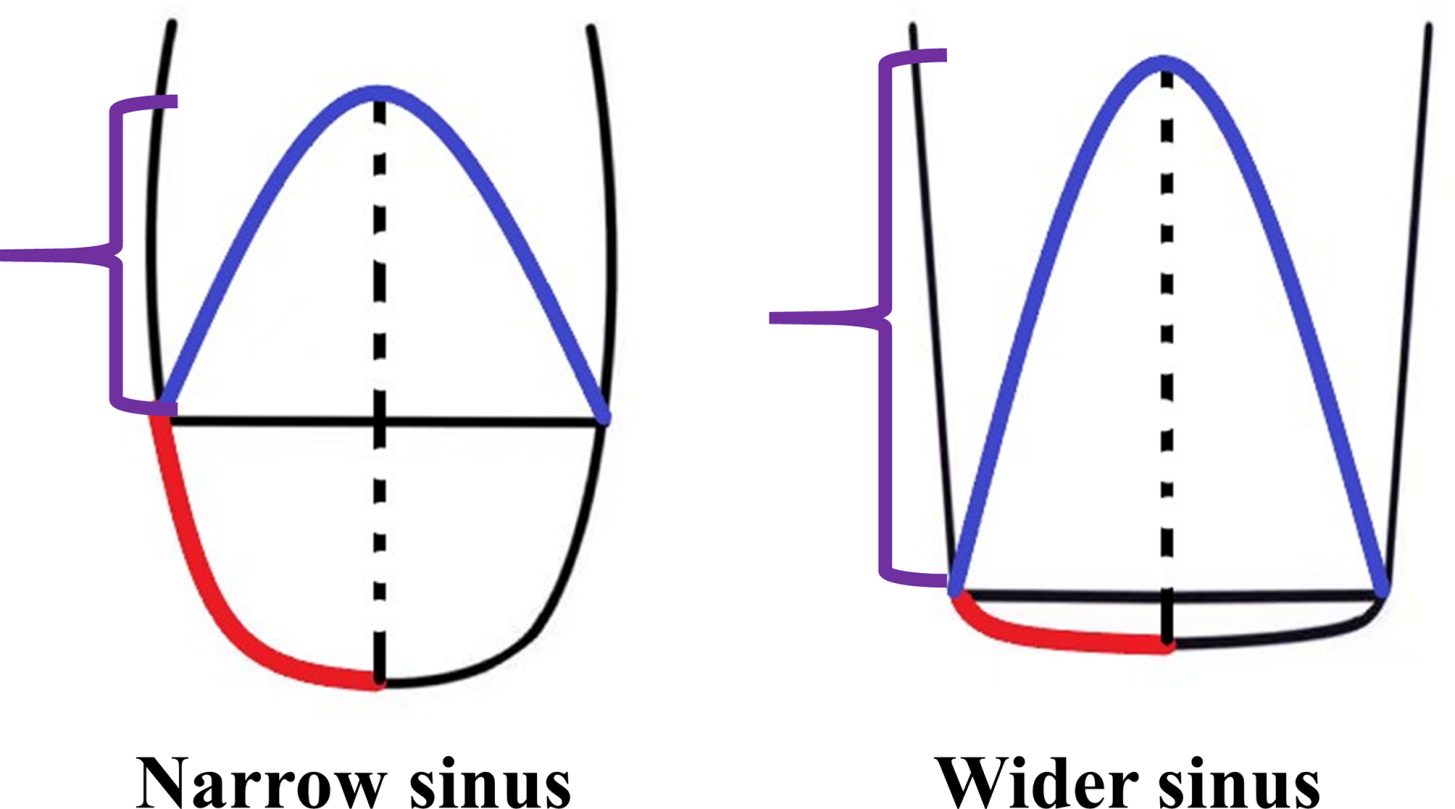
Schematic representation of the extent of membrane stripping and the height to which it can be elevated. The vertical distance to which the membrane is lifted by the tip of the implant (the height shown by the purple line and the portion of the membrane that is under tension shown by the blue line) is less in the narrower maxillary sinus (the red line shows the extent of the stripped mucosa) than in the wider maxillary sinus after membrane stripping

**Fig. 4:**
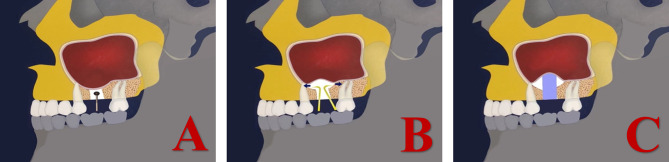
Illustrations of the modified internal sinus elevation procedure. (**A**) A Summer bone chisel was used to softly tap the alveolar crest, and this bone block was used as the roof of the area of elevated maxillary sinus floor. (**B**) Separation of the maxillary sinus floor membrane from the alveolar crest using a mucoperiosteal stripper. (**C**) Implant placement

**Fig. 5:**
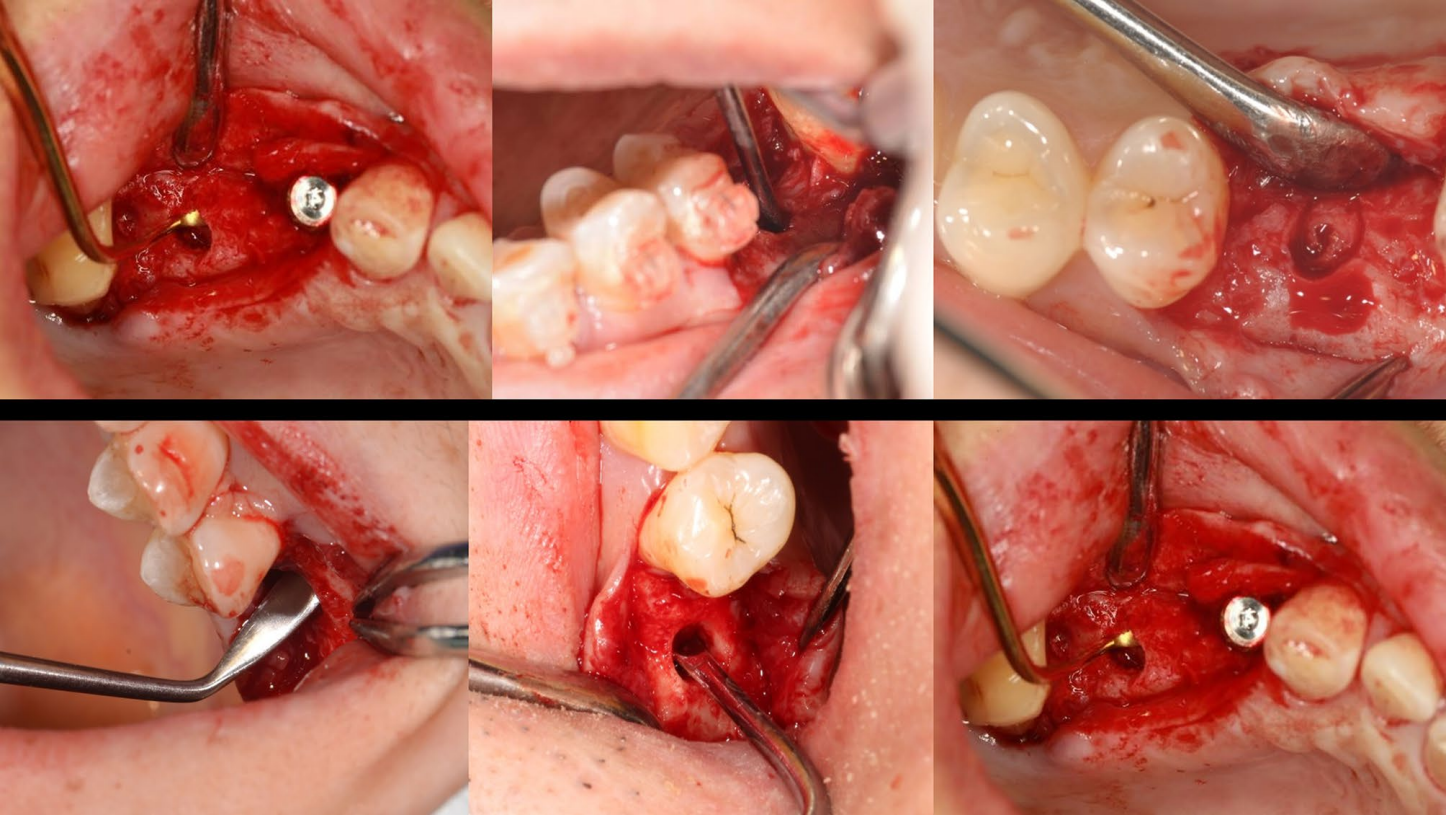
Separation of the maxillary sinus floor membrane/sinus mucoperiosteal detachment procedure

**Fig. 6:**
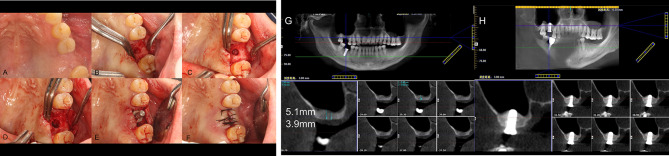
Surgical procedure for modified internal sinus elevation. (**A**) Preparation for surgery. (**B**) The alveolar crest is flat and slightly bluish. (**C**) Bone block serving as the roof of the area of elevated maxillary sinus floor. (**D**) Separation of the maxillary sinus floor membrane from the alveolar crest using a mucoperiosteal stripper. (**E**) Implant placement implant (the bone condensing technique was used to obtain good initial stability). (**F**) Tight suturing after implantation. (**G**) CBCT before surgery. (**H**) CBCT immediately after surgery

As noted in the previous paragraphs, the maxillary sinus was classified in terms of angle and width (Figs. 2 and 3) [36, 37]. Additionally, we collected 80 maxillary sinus data points from patients who underwent modified internal sinus elevation (Figs. 4, 5 and 6) at the Oral Implantation Center of Beijing Stomatological Hospital, affiliated with Capital Medical University (Beijing, China) between February 2020 and July 2020. All patients were informed about the surgical plan and possible complications and provided signed informed consent forms, agreeing to further analysis of their CBCT data. The protocol for this study was designed following the World Medical Association Declaration of Helsinki for biomedical research involving human subjects. The local ethics committee approved the study (number CMUSH-IRB-KJ-PJ-2018-06).

The CBCT data were divided into groups based on the various maxillary sinus angles, as shown in Table 1.

Maxillary sinus I: 45°, representing a narrow sinus.

Maxillary sinus II: 85°, representing a medium-width sinus.

Maxillary sinus III: 125°, representing a wide sinus.

**Table 1:** Maxillary sinus angle measurements

Maxillary sinus angle	Number
< 70°	5
70°— 85°	34
86°— 100°	36
> 100°	5

We chose the 85° maxillary sinus in the median group to represent the medium-width maxillary sinus. Moreover, we comparatively studied the stress distribution characteristics of the mucosa at the maxillary sinus floor between the narrower maxillary sinus and the wider maxillary sinus during the modified maxillary sinus elevation procedure. We also adjusted the angle of the maxillary sinus ourselves in Geomagic Studio 2014 software, creating 45° and 125° maxillary sinus models; to simplify the model, the two maxillary sinuses with their respective angles were constructed in a symmetrical bowl-shaped structure. After the modeling was finished, maxillary sinus grouping was performed as follows:

We used Mimics 21.0 software for data extraction to reconstruct 3D geometric models of the maxillary sinus and mucosa and exported the file in STL format. Next, an overall processed geometric model of the maxillary sinus and mucosa was obtained in STP format in Geomagic Studio 2014 software by patching, noise reduction, and surfacing. Subsequently, the STP file was imported into HyperMesh 14.0 software for meshing, and the BDF file was then exported for finite element mesh property setting, material parameter definition, load application, boundary condition constraint, and various computational working condition analyses via the finite element preprocessing software MSC Patran 2012 and the finite element postprocessing software MSC Nastran 2012 for the analysis of different computational working conditions.

**Fig. 7:**
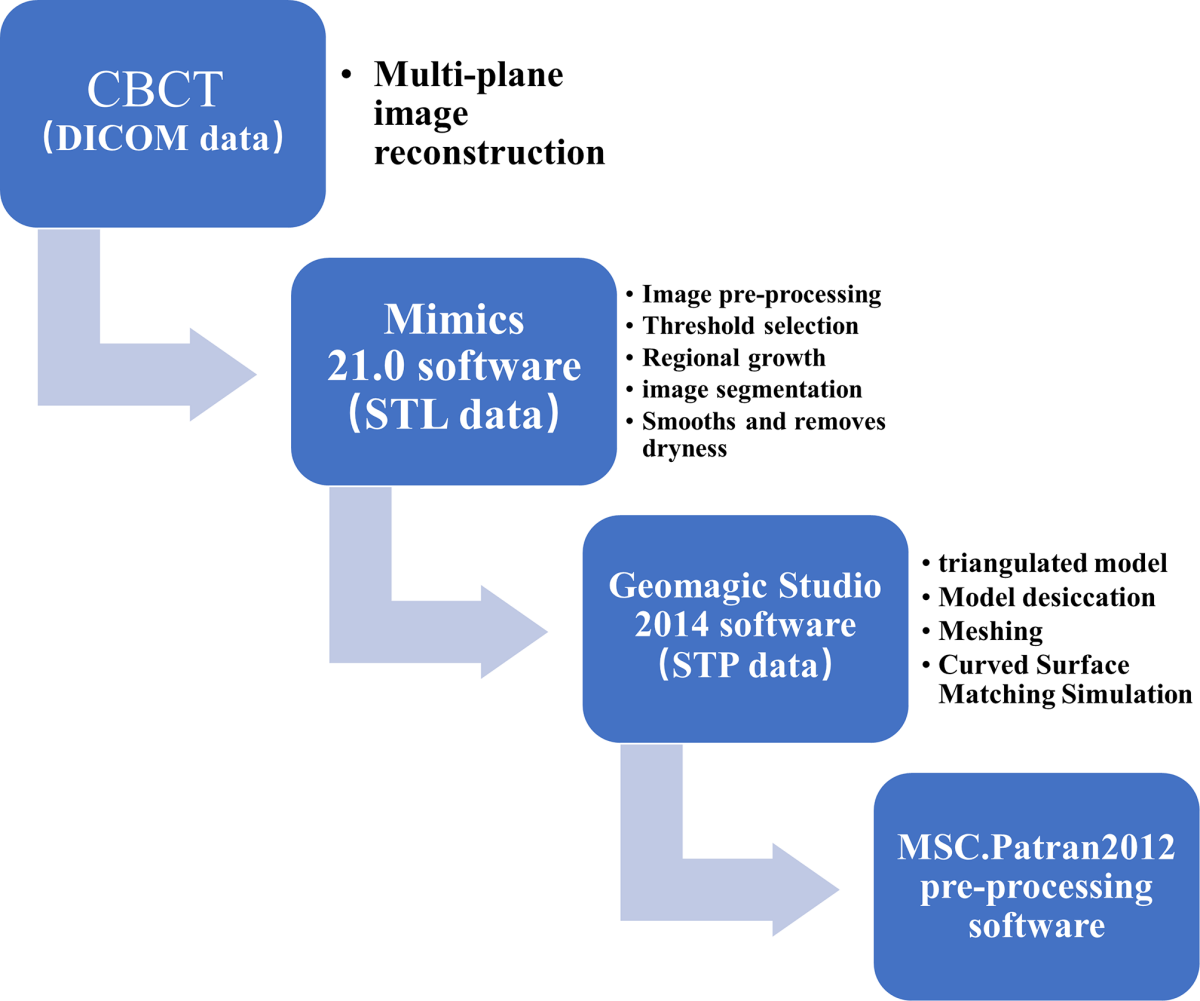
3D reconstruction model operation procedure

**Fig. 8:**
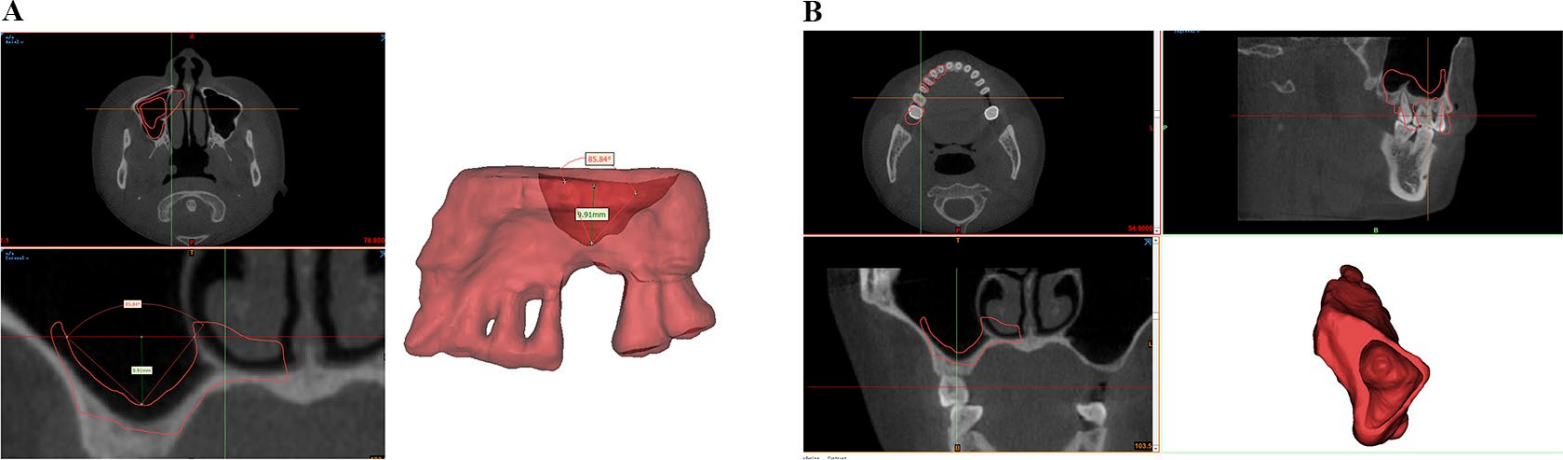
Schematic diagram of the 85° maxillary sinus as an example. Membrane CBCT data were extracted and then fitted to the bone model for matching and fitting

The process of manipulating the geometric model for 3D reconstruction of the maxillary sinus bone and membrane is shown in Figs. 7 and 8.

**Fig. 9:**
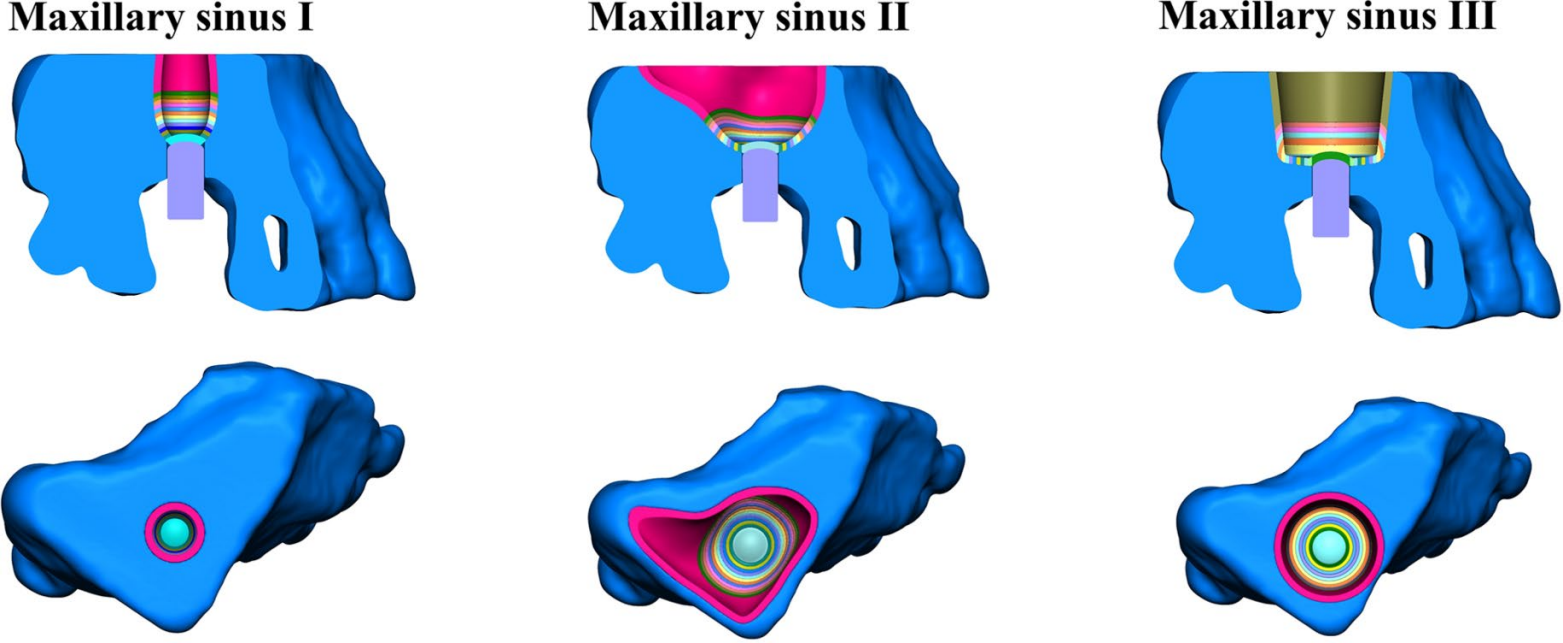
Reverse processing of solid geometric maxillary sinus bone and membrane models

The solid geometric models of the bone and membrane of the maxillary sinus at different angles were inverted and assembled with the geometric model of the implant, as shown in Fig. 9.

**Fig. 10:**
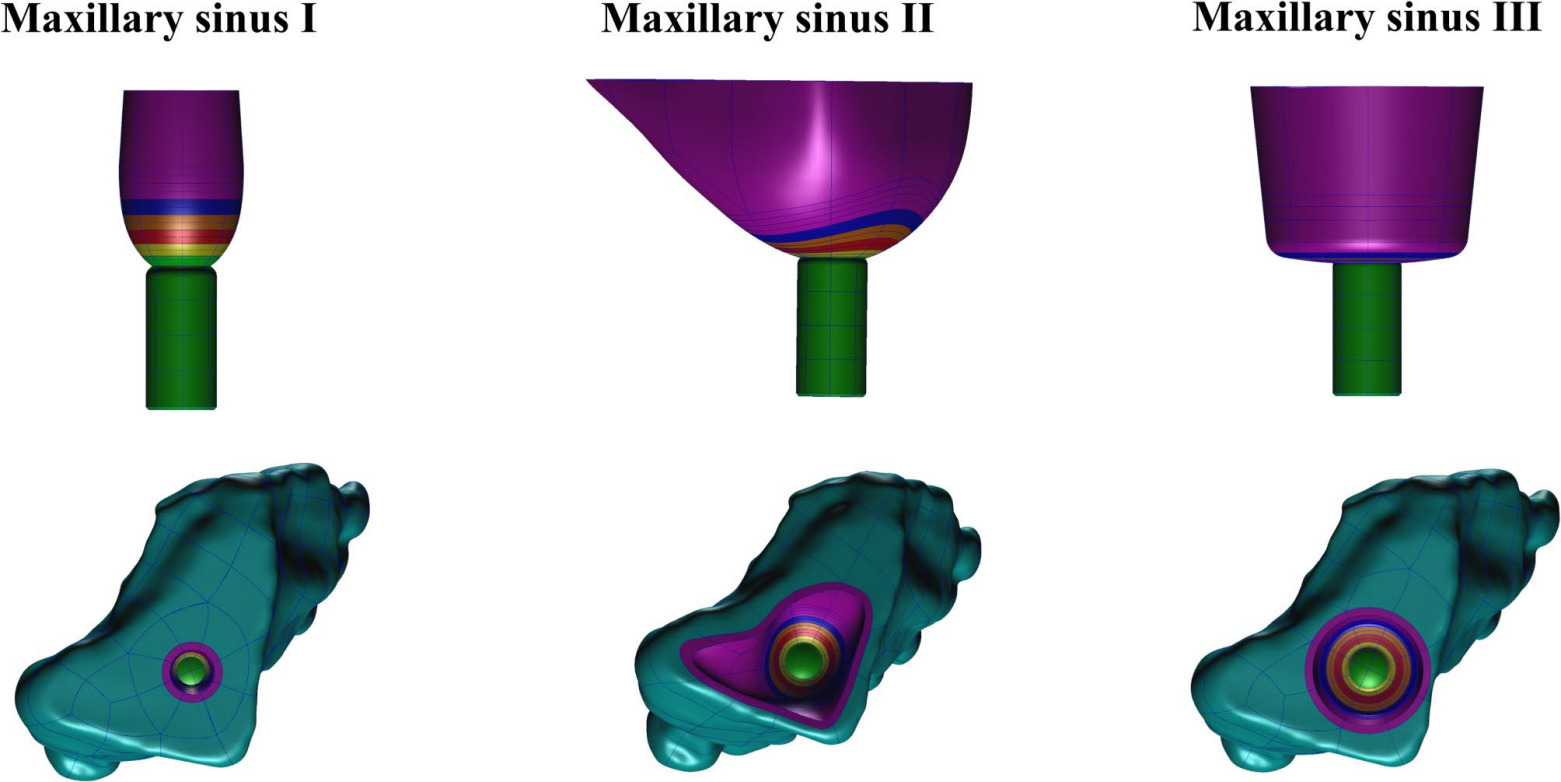
Geometric models imported into finite element preprocessing software

The geometric solid models corresponding to the three groups of maxillary sinus bones, membranes and implants were inverted and imported into MSC Patran 2012 software for structural assembly, as shown in Fig. 10.

### Finite element meshing

**Fig. 11:**
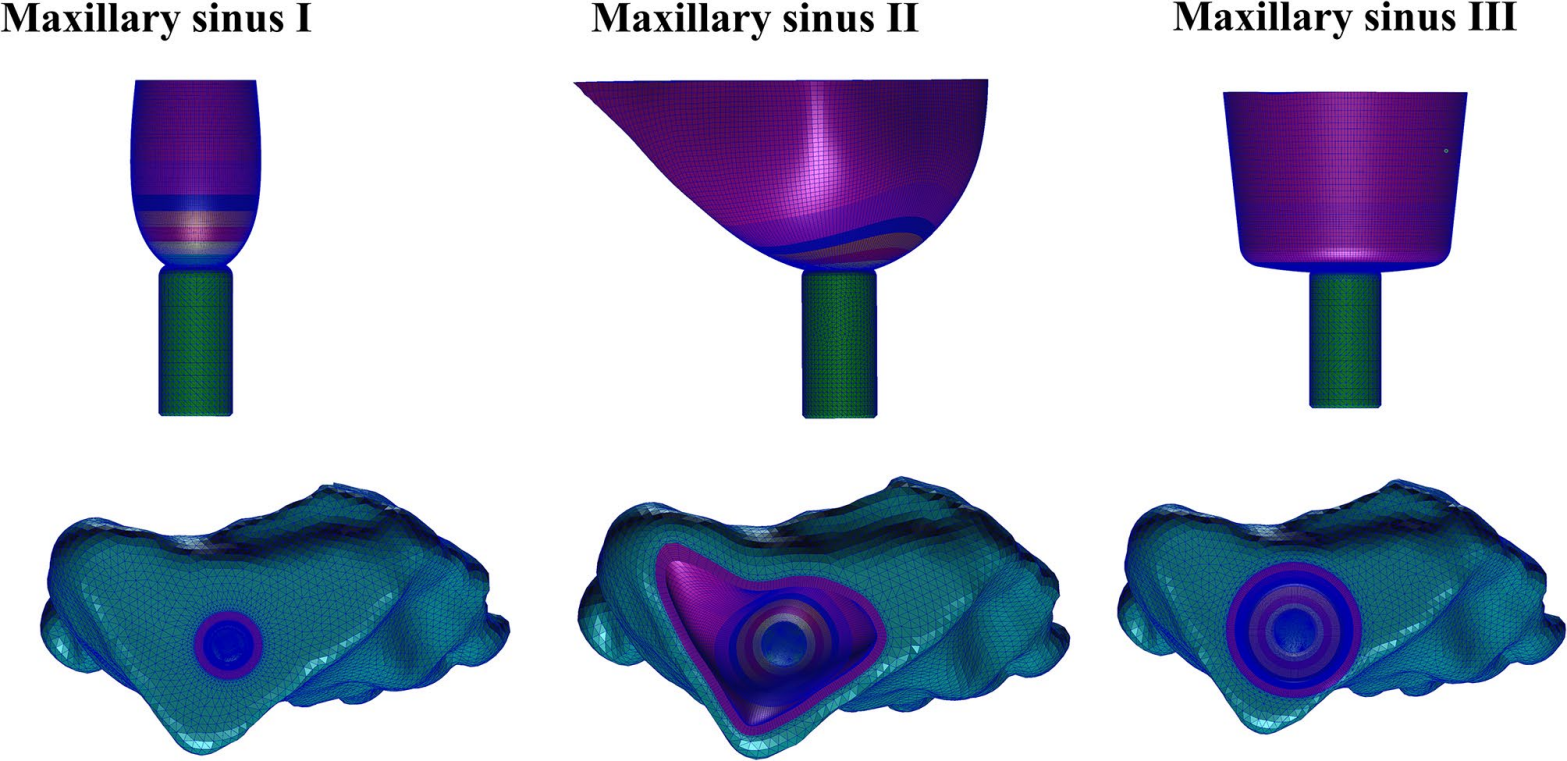
Finite element mesh maxillary sinus and membrane models imported into MSC Patran 2012

The STP files of the implant and corresponding maxillary and sinus membrane models were imported into HyperMesh 14.0 software (Altair Engineering, Inc., Troy, MI, USA) for meshing. The finite element mesh property settings, material parameter definitions, loading conditions, and boundary condition constraints were applied in MSC Patran 2012 software, as shown in Fig. 11.

The parameters used to mesh the finite element model of the maxillary sinus membrane at three different angles are shown in Table 2. The maxillary sinus bone, membrane, and implant were all meshed with solid units, and to improve the calculation accuracy, convergence, and efficiency, the membrane was divided into hexahedral mesh units (IsoMesh Hex8 Element), and the implant and maxilla were divided into tetrahedral mesh units (TetMesh Tet4 Element).

**Table 2:** Finite element model mesh parameters for maxillary sinus membrane at three different angles

	Maxillary sinus I	Maxillary sinus II	Maxillary sinus III
Nodes	295,809	361,514	590,735
Elements	563,688	367,466	550,882

### Biomechanical properties

The mechanical properties of the material for each structure are shown in Table 3.

**Table 3:** Mechanical properties [39] of materials for the maxilla, sinus membrane, and implant structures

Materials	Elastic modulus (MPa)	Poisson’s ratio
Cortical bone of the maxilla	13,700	0.30
Cancellous bone of the maxilla	1370	0.30
Sinus membrane	Hyperelastic C_10_ = 0.253, C_01_ = 0.027	
Implant (titanium)	110,000	0.35

**Fig. 12:**
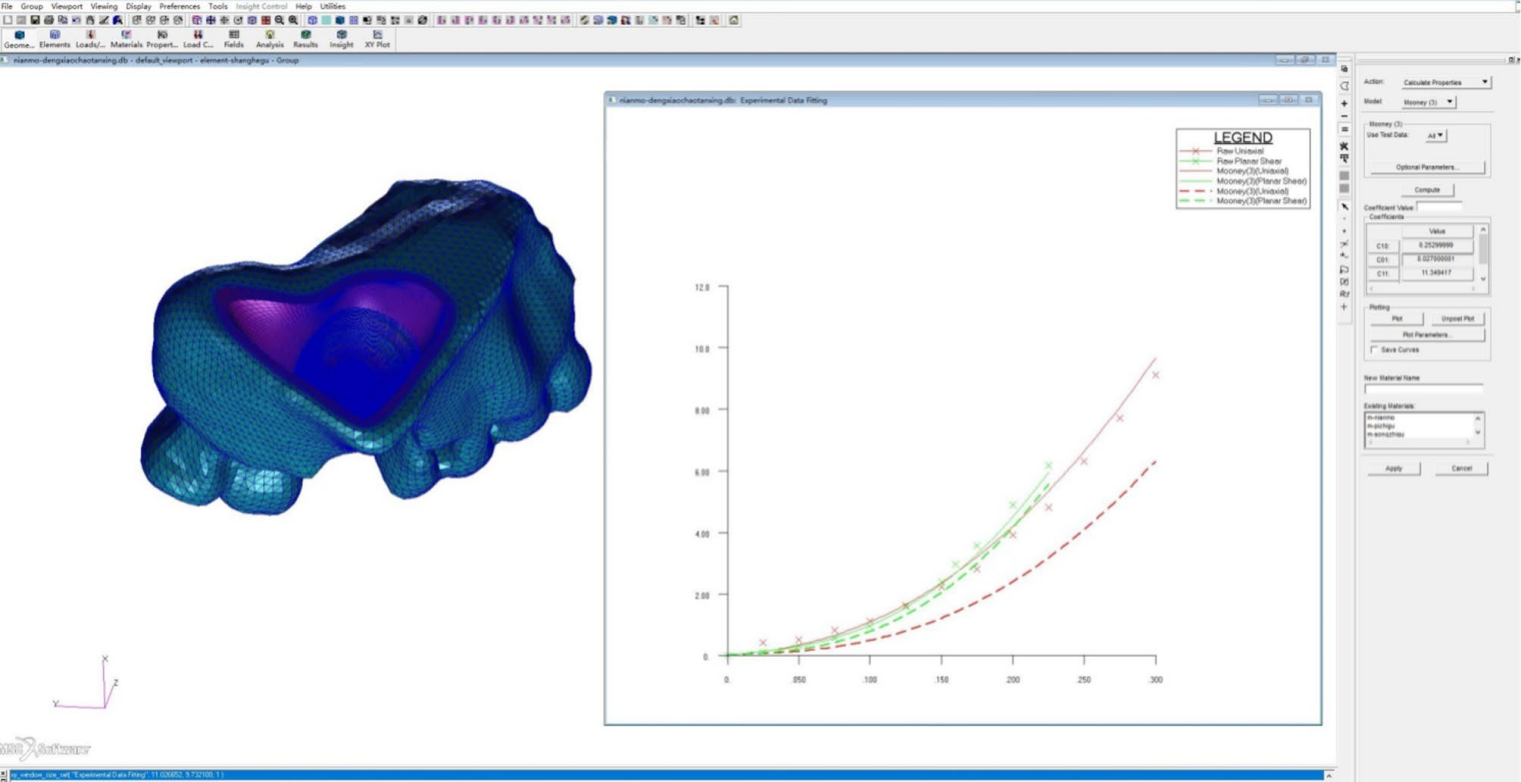
Hyperelastic material parametric equivalent fitting of the maxillary sinus membrane

The maxillary sinus membrane is a large-deformation nonlinear soft tissue with hyperelastic mechanical properties; thus, nonlinear, large-displacement deformation of the complex material is needed to allow the computation to be equivalent to the maxillary sinus membrane in terms of hyperelastic properties. The equivalence method is briefly described, as follows:

Considering the approximately isotropic, incompressible, hyperelastic material properties of the maxillary sinus membrane, which can be expressed analytically by the associated stress‒strain relationship, the measured data can be fitted to obtain the material parameters. A typical stress versus stretch form of a Mooney-Rivlin model for hyperelasticity was chosen for the curve fitting to evaluate the material parameters, where C10 and C01 are two typical material parameter coefficients for a hyperelastic Mooney–Rivlin model. Experimental data fitting was achieved via the Tools–Modeling–Experimental Data Fitting function in the MSC Patran/Nastran 2012 software. The measured stress‒strain curve data of the relevant materials were input through the Select Test Data command, and finally, the Mooney–Rivlin type was selected to calculate the properties for equivalent fitting; the process of equivalent fitting is shown in Fig. 12.

### Loading and constraint conditions

The material assumptions, boundary limitations, and loading conditions of the structures were as follows:

- Nodes that constrained the surface of the maxilla were fixed, limiting their freedom of motion in three directions.
- The maxilla and implants were assumed to be isotropic, homogeneous, and continuous linear elastic materials, and the sinus membrane was equivalent to a hyperelastic material (nonlinearity, large displacement, and large deformation).
- During implant movement, only displacement in the direction of the pushing height was permitted; lateral displacement was restricted.
- When different degrees of membrane separation were simulated, the unseparated membrane region was considered the common node, while the membrane of the separated region was not in contact with the node of the corresponding maxillary region and was not considered the common node.
- The implant tip was considered to have frictional contact with the bottom of the membrane, the implant periphery was not in contact with the maxillary cavity preparation region, and the separated and nonseparated membranes were processed as a common node.

### Experimental model groupings

**Fig. 13:**
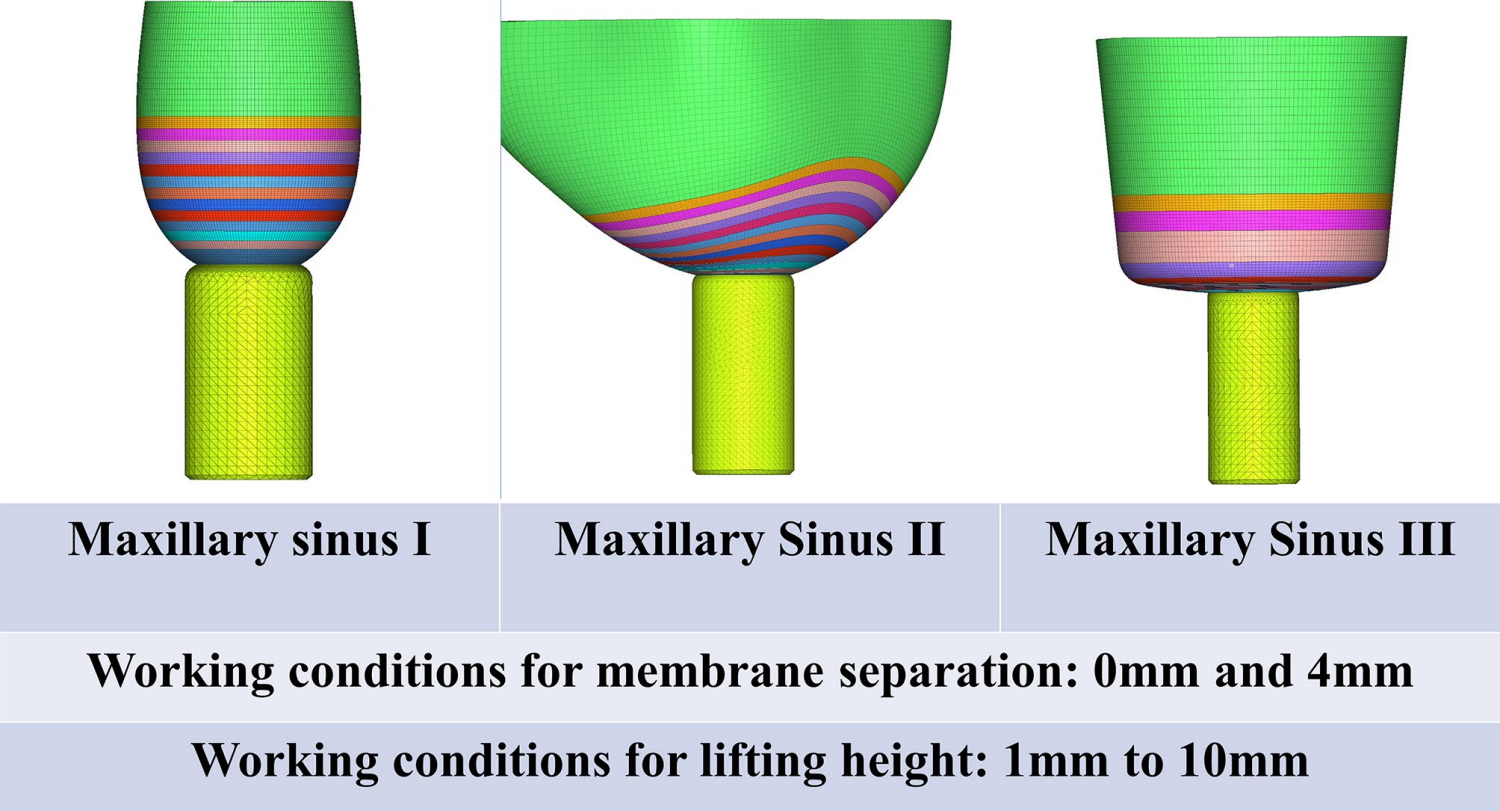
Experimental model groupings

The implants were elevated to different heights (1–10 mm) at three different maxillary sinus angles. The membrane of the maxillary sinus floor was also stripped by 0 and 4 mm, representing the traditional internal sinus elevation procedure and the modified internal sinus elevation procedure that we performed, respectively. The experimental model groupings are shown in Fig. 13.

### Finite element analysis

**Fig. 14:**
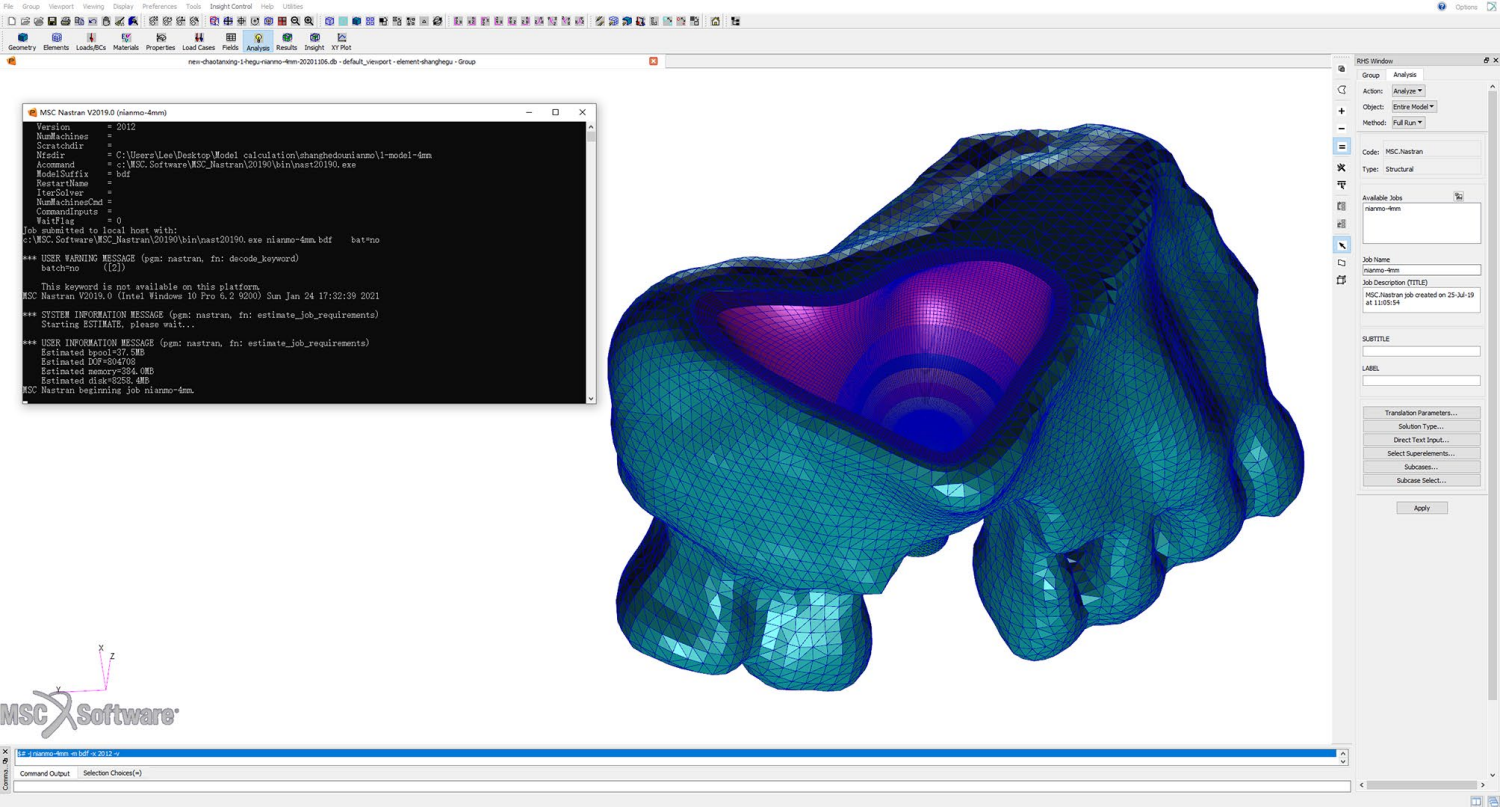
Finite element analysis computational software screen

The finite element simulation analysis was performed on models under various working conditions before postprocessing in MSC Nastran 2012 software (NASA, USA), which was used for computational analysis and visualization of the results. The stress distribution nephogram and peak stress of the maxillary sinus membrane structure were obtained for each model group. The primary observation indicator was the value of the equivalent von Mises stress of the maxillary sinus floor membrane. The computational software is shown in Fig. 14.

## Results

For models under each working condition, the stress distribution nephogram of the maxillary sinus floor membrane was observed from the inferior view, the peak stress was calculated, the features of the stress distribution were examined, and graphs were created to compare the peak membrane stress values of each group of models.

**Fig. 15:**
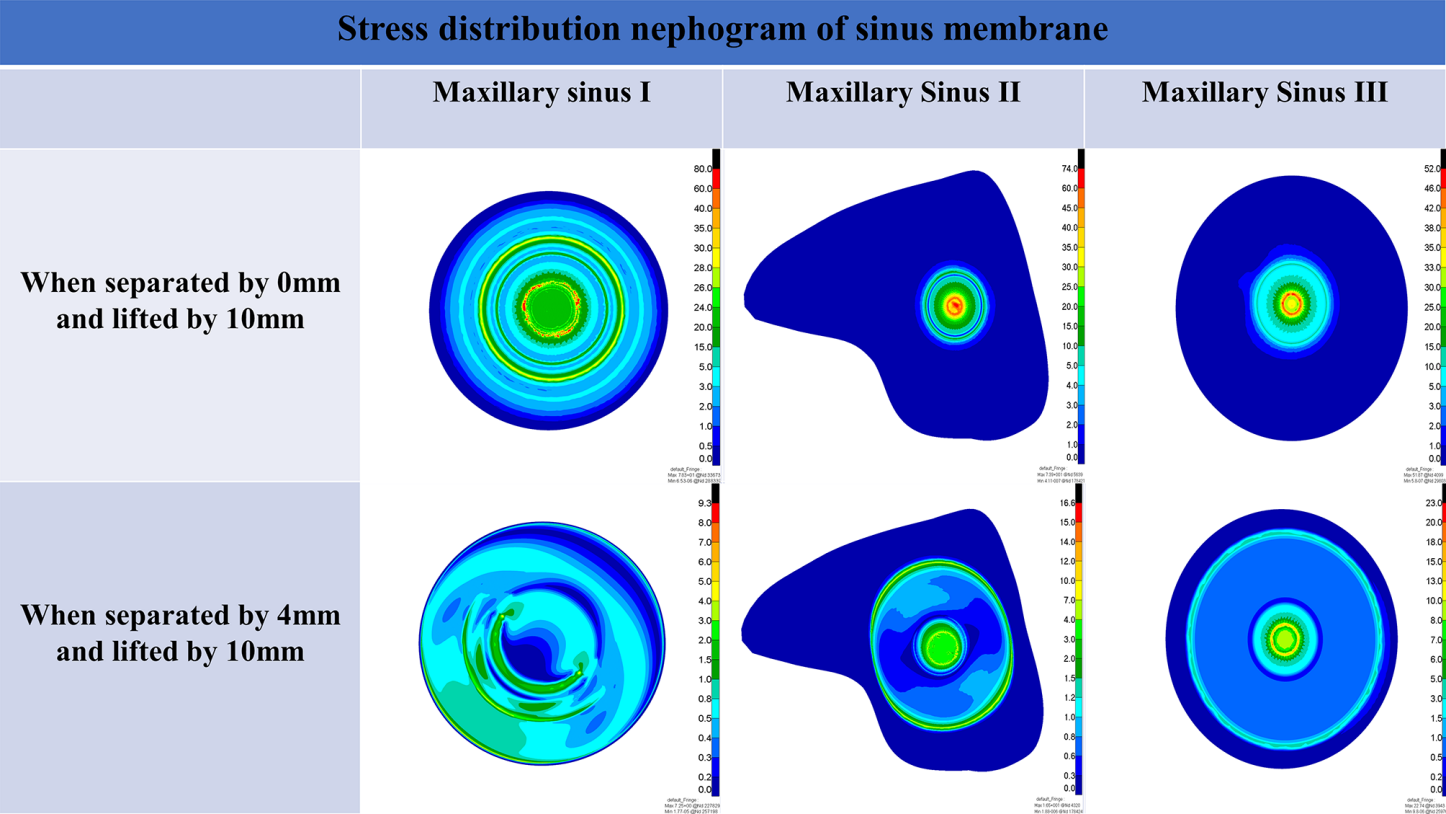
Stress distribution nephograms of the sinus membrane

The nephograms (Fig. 15) illustrate the three different angles of the maxillary sinus floor membrane stress distribution with an elevation height of 10 mm and 0 mm and 4 mm degrees of sinus membrane separation.

**Fig. 16:**
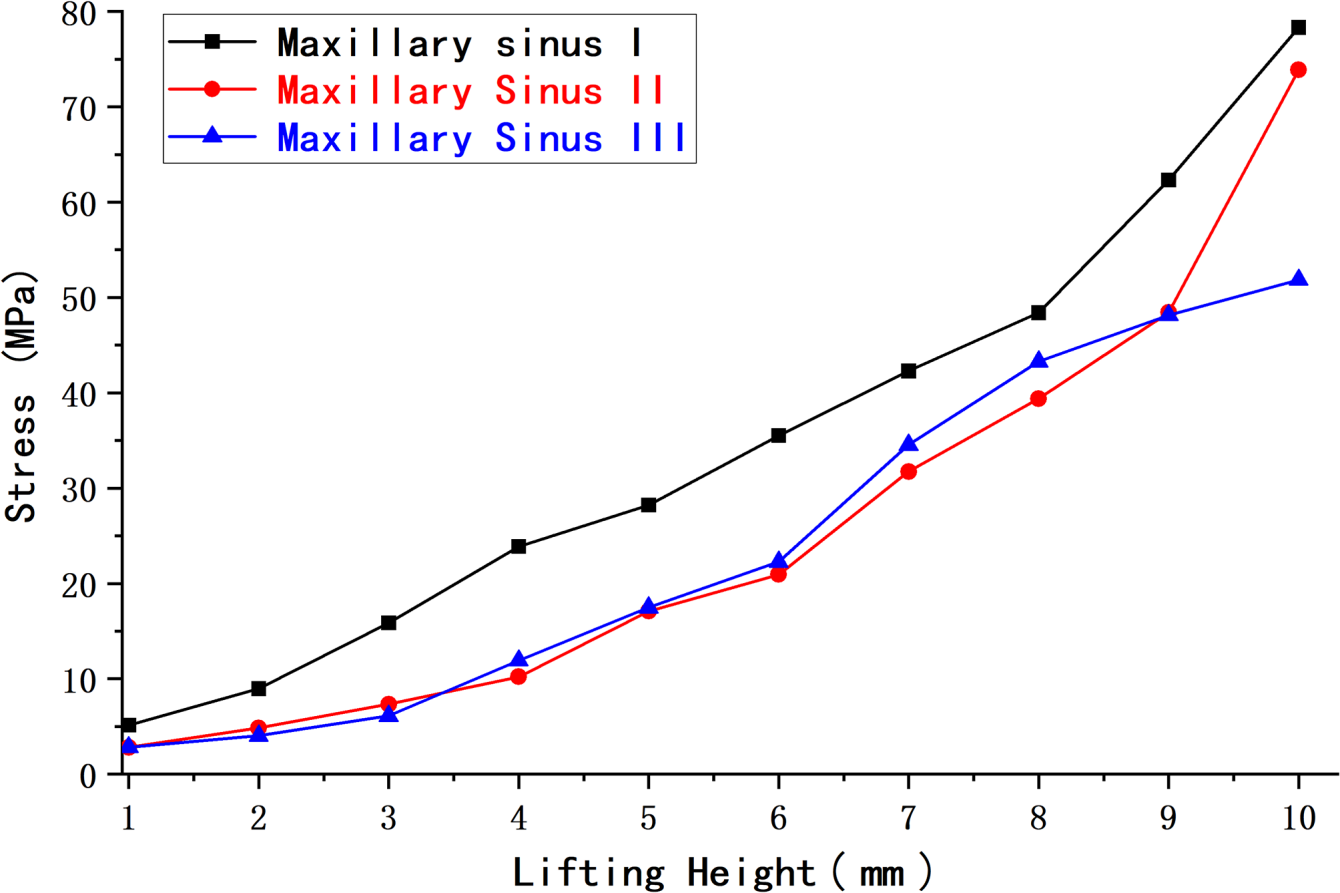
Peak maxillary sinus floor membrane stress at 0 mm of separation for three working conditions

1. The peak membrane stresses (MPa) at three different maxillary sinus angles and 0 mm of maxillary sinus floor membrane separation are shown in Table 4; Fig. 16.

**Table 4:** Peak membrane stress (MPa) at 0 mm of separation for three different maxillary sinus angles

Elevation height (mm)	Maxillary sinus I	Maxillary sinus II	Maxillary sinus III
1	5.14	2.81	2.82
2	8.97	4.84	4.03
3	15.88	7.34	6.12
4	23.88	10.21	11.92
5	28.22	17.10	17.47
6	35.51	20.94	22.27
7	42.29	31.73	34.54
8	48.40	39.40	43.28
9	62.33	48.42	48.16
10	78.32	73.89	51.87

**Fig. 17:**
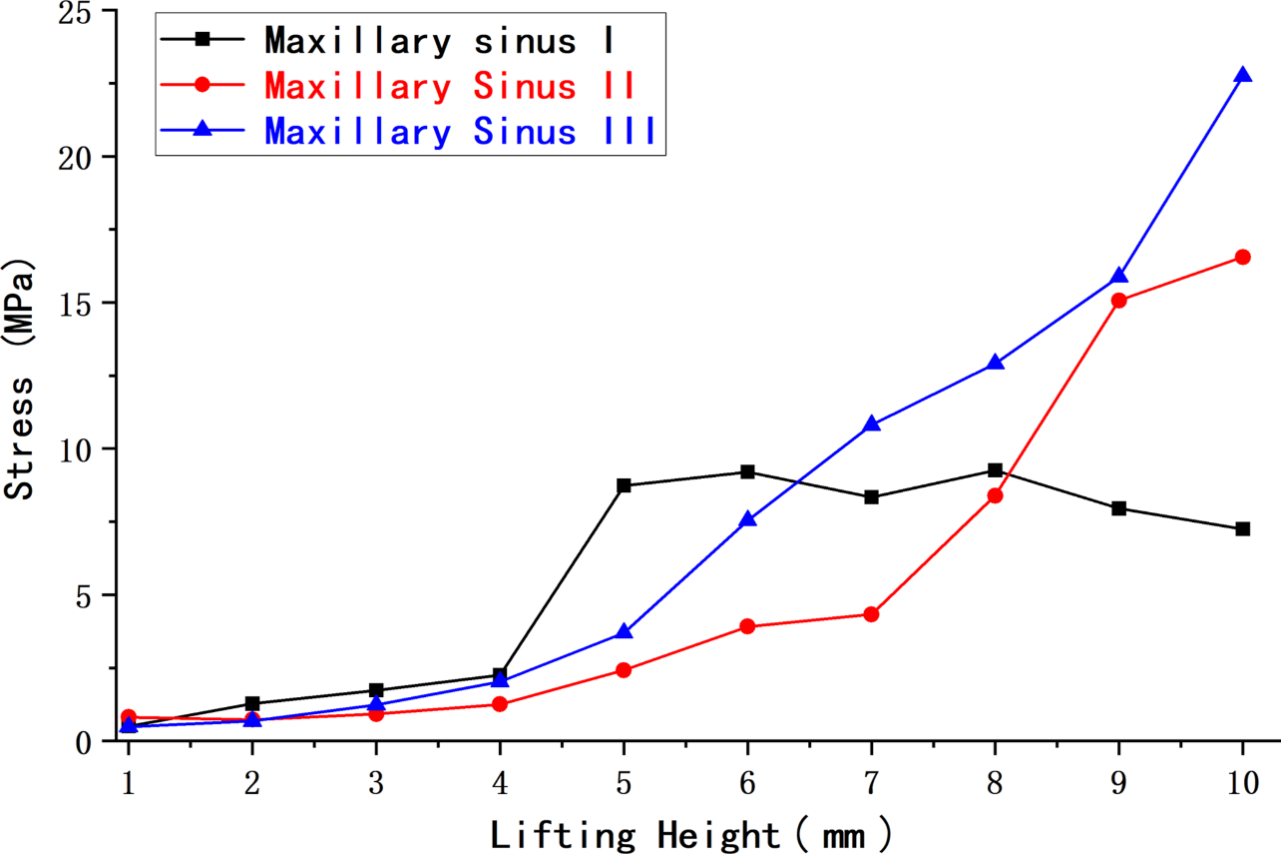
Peak maxillary sinus floor membrane stress at 4 mm of separation for three working conditions

2. The peak membrane stresses (MPa) at three different maxillary sinus angles and 4 mm of maxillary sinus floor membrane separation are shown in Table 5; Fig. 17.

**Table 5:** Peak membrane stress (MPa) at 4 mm of separation for three different maxillary sinus angles

Elevation height (mm)	Maxillary sinus I	Maxillary sinus II	Maxillary sinus III
1	0.50	0.81	0.49
2	1.27	0.73	0.68
3	1.73	0.92	1.24
4	2.26	1.25	2.03
5	8.74	2.42	3.70
6	9.20	3.91	7.55
7	8.34	4.33	10.81
8	9.26	8.39	12.91
9	7.95	15.07	15.88
10	7.25	16.55	22.74

**Fig. 18:**
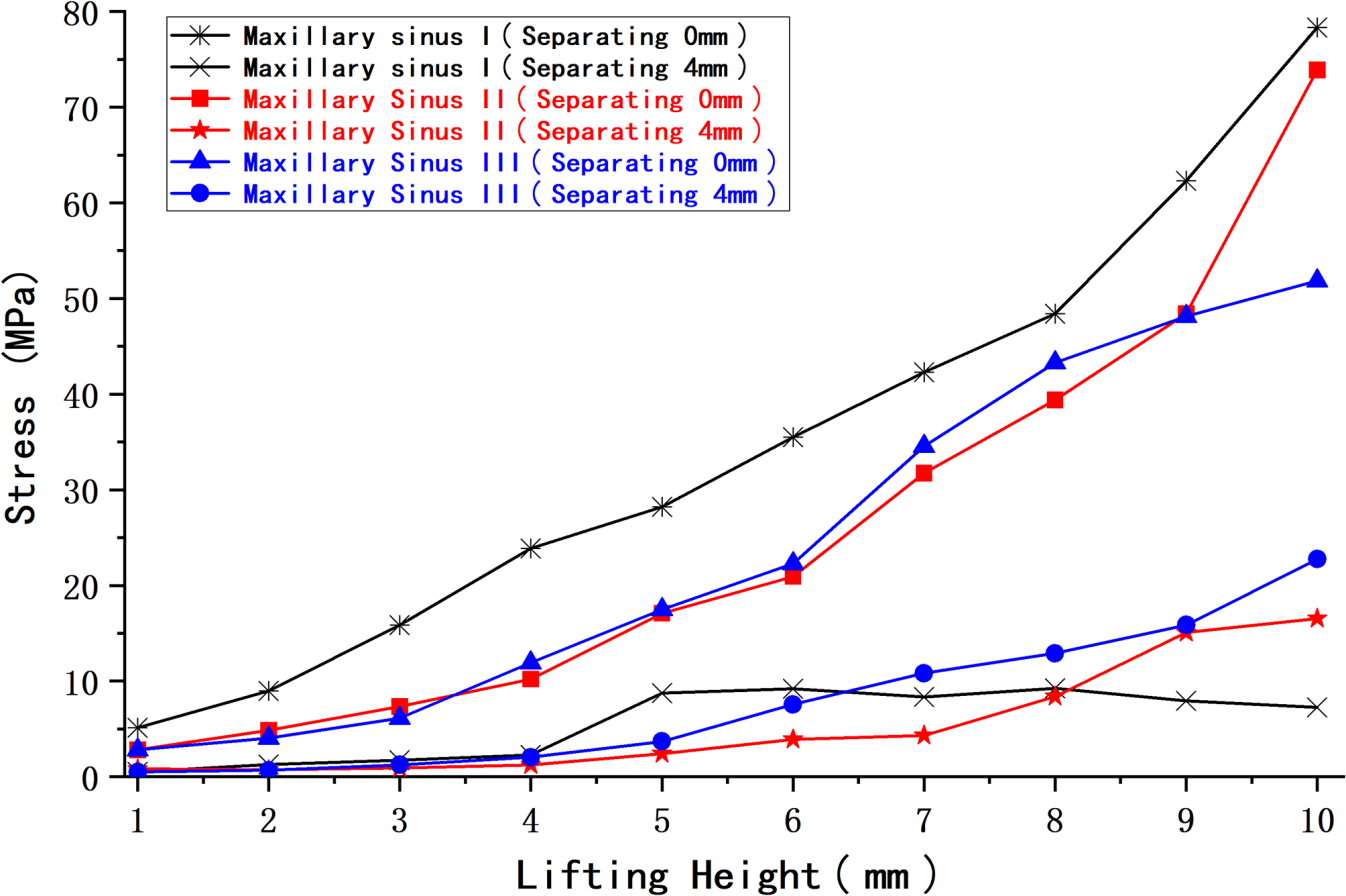
Peak maxillary sinus floor membrane stress at 0 and 4 mm of separation for three working conditions

Figure 18 shows a comparison of the maxillary sinus stress at 0 and 4 mm of sinus floor membrane separation and three different maxillary sinus angles.

## Discussion

Given the results of this finite element analysis, we were able to outline some useful recommendations regarding the preoperative planning of internal sinus elevation, the radiographic assessment and the sinus mucoperiosteal separation procedure.

### Experimental methodology and parameter design

The finite element method [40] regards the research object as a continuous elastic unit. This unit is decomposed into a series of minor finite object units, that is, finite elements, which then forms an aggregate of units to replace the original continuum and allow the overall stress distribution to be examined by studying the properties of each unit one by one. Therefore, the method can reflect the stresses, strains, and displacements in each part of the structure, especially the inner parts. The finite element method has progressed from simple 2D structural analysis to complex 3D structural analysis methods and has been widely applied in oral biomechanics [41, 42]. Through in vitro experiments, we have learned about the mechanical and deformation characteristics of the maxillary sinus membrane [43, 44]. Perforation of the membrane occurs only once the pressure applied during the peeling process exceeds the membrane’s acceptance constraint, as determined in the previously mentioned in vitro experiments. The risk of perforation can be avoided only by limiting the height of elevation and precisely assessing anatomical locations because it is challenging to manage the force of punching and peeling during the actual procedure. Although there have been few published studies [39] on the material constitutive and failure strength of the maxillary sinus membrane, determining the actual failure strength requires many cadaveric tests and relevant theoretical research. Therefore, we used the finite element analysis method to calculate the equivalent hyperelastic properties of the maxillary sinus mucosa through matching to obtain more similar hyperelastic C10 and C01 parameters; then, we input them into the finite element software for calculation and analysis from a qualitative point of view to study the force characteristics of the mucosa on the maxillary sinus.

The absolute values of the finite element calculation results are hardly representative of the actual values of the human body. However, if the clinical operation is correctly simulated, under the assumptions of the boundary conditions, simulated loading conditions, and impact loading conditions, the stresses and displacements generated at the interface show the same trend, and the results can play a specific role in guiding clinical operations. In this study, the method of impact loading was used, the calculations were smooth, and converged results were obtained. The trend of the stress and displacement values at the interface indicates that our model shows geometrically solid and biomechanical similarities to in vivo conditions and that the design of the experimental parameters is reasonable and can provide guidance for clinical operations. However, further research is still needed to create a computational model that completely replicates the intricate oral biomechanical environment.

### Modeling basis and geometric accuracy of the 3D finite element model

To simulate internal sinus elevation as realistically as possible, we used the preoperative CBCT data of patients who had already undergone the modified internal sinus elevation procedure in the clinical setting for accurate modeling. Moreover, because of the diversity of maxillary morphology, in this study, we qualitatively compared only the stress, strain, and displacement of the maxillary sinus floor membrane during the rotational insertion of the implant. We also simplified the model to a certain extent by simulating only the bone in the maxillary sinus region instead of the intact maxilla, which did not affect the comparison of the results. In finite element analysis, models are commonly simplified by omitting secondary aspects for complex entities and making certain assumptions for test conditions.

The thickness of the maxillary sinus membrane exhibits considerable interindividual differences. Many researchers [45–47] have measured and studied the thickness of the maxillary sinus membrane. Most scholars [48, 49] have used CBCT data in their work, while a few have measured mucosal tissue directly from cadaveric specimens. CBCT measurement errors can cause imaging measurements to be greater than those obtained from cadaveric specimens. Ongoing discussion exists on a standard imaging threshold for the maxillary sinus mucosal thickness. With most studies using 1 mm as a standard [50, 51], we simplified the maxillary sinus membrane by setting its thickness to 1 mm.

While lifting the maxillary sinus membrane with internal sinus elevation, we concentrated on maxillary sinus floor membrane perforation. To investigate and compare the test results under different working conditions, we focused on the maxillary sinus membrane under the assumption of boundary conditions after assembling and meshing the geometric model. This test also allowed the analysis of changes in the biomechanical properties of the maxillary sinus membrane with different degrees of separation from a qualitative standpoint. This calculation and analysis may also support the accuracy of our modified internal sinus elevation model.

### Comparison of peak stresses in the membrane of the maxillary sinus floor under different working conditions

The above experimental results revealed that at 0 mm of maxillary sinus floor membrane separation, with increasing elevation, the maxillary sinus floor membrane in the three corresponding maxillary sinus angle groups showed a nonlinear increase in force. However, the magnitude and amplitude of the increase varied. At different internal elevation heights, the peak membrane stress of maxillary sinuses II and III was notably lower than that of maxillary sinus I, with decreases ranging from approximately − 57.2% to -5.7% and − 61.5% to -10.6%, respectively. For both maxillary sinuses II and III, the stress of the maxillary sinus floor membrane at 4 mm of separation gradually increased with increasing elevation, whereas the corresponding stress of maxillary sinus I showed an increasing and then slowly decreasing trend. At an elevation height of 6 mm or less, the peak membrane stress of maxillary sinuses II and III was lower than that of maxillary sinus I, with decreases of approximately − 72.3% to -9.4% and − 57.7% to -2.0%, respectively. At an elevation height of 6.5 mm or more, the peak membrane stress of maxillary sinus III was greater than that of maxillary sinuses I and II. The increase in membrane stress compared to that in maxillary sinus I was approximately 29.6–213.7%, while the increase in membrane stress in maxillary sinus II compared to that in maxillary sinus I at an elevation height of 8 mm or more was approximately 89.6–128.3%. These characteristic changes in stress may be related to the morphological changes in the mucosal structure caused when separating the mucosa of the floor of the narrower maxillary sinus.

We discovered variations in the efficiency of maxillary sinus floor elevation with different angles and morphologies, in addition to the effects of the different morphologies (shallow concave, deep concave, and convex) on the stress and displacement of the sinus floor membrane that other scholars [52] have previously studied. Here, elevation efficiency can be understood as the amount of stress on the mucosa of the maxillary sinus floor during elevation, with less stress when elevating to the same height indicating greater efficiency. At 0 mm of separation, a deep concave sinus floor approximately corresponds to maxillary sinuses II and III defined in this study, and a shallow concave sinus floor approximately corresponds to maxillary sinus I. These results show that at the same elevation height, the peak mucosal stress on the floor of both maxillary sinuses II and III is less than that of maxillary sinus I. We did not specifically model a convex sinus floor. However, in conjunction with our previous analyses, the present results suggest that the morphology of the sinus floor of maxillary sinus I is similar to that of a convex sinus floor with 4 mm of membrane separation and an elevation of more than 6 mm. When exposed to the impact, the convex maxillary sinus floor shows a more substantial stress dispersion effect. This also explains why the peak membrane stress of maxillary sinus I decreases beyond an elevation height of 6 mm at 4 mm of separation.

The peak membrane stress on the sinus floor at 4 mm of separation was lower than that at 0 mm for the same maxillary sinus under various operating conditions. Additionally, the nephogram indicates that the stress concentration quickly increases in the region where the implant tip is in contact with the membrane and intensifies with increasing elevation. The stress concentration phenomenon improved when the membrane was at 4 mm of separation. Therefore, sufficient decortication of the maxillary sinus membrane will improve the stress concentration and reduce the risk of rupture. This means that stripping the sinus floor membrane is straightforward and effective, and our modification is reliable and relatively safe.

## Conclusions

With traditional internal sinus elevation, the peak membrane stress of maxillary sinus I was notably greater than that of maxillary sinuses II and III at different elevation heights. This means that the risk of rupture and perforation of the membrane at the floor of the narrow maxillary sinus was relatively high without separation of the floor membrane. In the modified internal sinus elevation procedure, the membrane stress of maxillary sinuses II and III tended to increase as the elevation height increased, whereas the stress of maxillary sinus I tended to increase and then slowly decrease. In other words, after separating the membrane of the sinus floor, the risk of rupture and perforation of the membrane of the narrow maxillary sinus was relatively low with greater elevation. In summary, the risk of maxillary sinus floor membrane rupture is greatly reduced after adequate stripping of the sinus floor membrane when performing modified internal sinus elevation in a narrow maxillary sinus. In wider maxillary sinuses, the risk of membrane rupture and perforation at the same elevation is relatively low, regardless of whether traditional or modified internal sinus elevation is performed.

### Electronic supplementary material

Below is the link to the electronic supplementary material.

Supplementary Material 1

Supplementary Material 2

Supplementary Material 3

Supplementary Material 4

Supplementary Material 5

Supplementary Material 6

Supplementary Material 7

## Data Availability

The datasets used in the present study are available from the corresponding author upon reasonable request.

## Abbreviations

RBH: residual bone height
3D: three-dimensional
CBCT: cone-beam computed tomography
STL: stereolithography
BDF: bitmap distribution format
