# Supplementary material for: Biomechanical analysis of the maxillary sinus floor membrane during internal sinus floor elevation with implants at different angles of the maxillary sinus angles
Source: Int J Implant Dent. 2024 Mar 12;10:11. doi: 10.1186/s40729-024-00530-5 (PMC10933249; doi:10.1186/s40729-024-00530-5)
Supplement: Supplementary file 1 — Supplementary Material 1 [file 40729_2024_530_MOESM1_ESM.pdf]

首都医科大学附属北京口腔医院伦理委员会（科研/技术分会）

伦理审查批件

**审查批件号**	CMUSH-IRB-KJ-PJ-2018-06		
**项目名称**	对 1-4mm 上颌窦底高度的牙种植采用改良经牙槽嵴顶上颌窦提升手术的临床效果研究		
**项目编号**	17-09-20		
**项目来源**	院级课题-临床专项		
**研究单位**	首都医科大学附属北京口腔医院		
**项目负责人**	马攀		
**受理号**	KJ-2017-010-B-02-FS(CS)		
**审查类别**	复审（初始审查后）	**审查方式**	快速审查
**审查日期**	2018.3.30/4.2	**审查地点**	/
**审查委员**	江青松、郑宇同		
**批准文件**	1. 临床研究方案（版本号 2.0，日期：2018.1.10）<br>2. 研究病历（版本号 1.0，日期：2017.7.21）<br>3. 知情同意书（版本号 2.0，日期：2018.1.10）		

<table>
<tr><td>审查意见</td><td colspan="3">1. 根据 CFDA《药物临床试验伦理审查工作指导原则》(2010)、《药物临床试验质量管理规范》(2003)、《医疗器械临床试验质量管理规范》(2016)、WMA《赫尔辛基宣言》、CIOMS《人体生物医学研究国际道德指南》、国家卫生计生委《涉及人的生物医学研究伦理审查办法》(2016)的伦理原则，经本伦理委员会审查，同意按所批准的临床研究方案、知情同意书、招募材料开展本项研究。

2. 请遵循 GCP 原则、遵循伦理委员会批准的方案开展临床研究，保护受试者的健康与权益。

3. 研究过程中若变更主要研究者，对临床研究方案、知情同意书、招募材料等的任何修改，请负责人提交修正案审查申请。

4. 发生严重不良事件，请负责人及时提交严重不良事件报告。

5. 请按照伦理委员会规定的年度/定期跟踪审查频率，申请人在截止日期前 1 个月提交研究进展报告；当出现任何可能显著影响试验进行、或增加受试者危险的情况时，请负责人及时向伦理委员会提交书面报告。

6. 重大违背方案（研究纳入了不符合纳入标准或符合排除标准的受试者，符合中止试验规定未让受试者退出研究，给予错误治疗或剂量，给予方案禁止的合并用药等没有遵从方案开展研究的情况；或可能对受试者的权益/健康以及研究的科学性造成不良影响等违背 GCP 原则的情况）、持续违背方案，或对违规事件不予以纠正，请负责人提交违背方案报告。

7. 暂停或提前终止临床研究，请负责人及时提交暂停/终止研究报告。

8. 完成临床研究，请负责人提交研究完成报告。</td></tr>
<tr><td>年度/定期
跟踪审查频率</td><td>12 个月</td><td>有效期至</td><td>2019. 3. 31</td></tr>
<tr><td>主任委员签字</td><td>[signature]</td><td>日期</td><td>2018 年 4 月 9 日</td></tr>
<tr><td>或指定授权人签字</td><td></td><td>日期</td><td>年 月 日</td></tr>
<tr><td>伦理委员会（盖章）</td><td colspan="3">首都医科大学附属北京口腔医院 伦理委员会</td></tr>
<tr><td>联系方式</td><td colspan="3">地址：北京口腔医院办公楼 508 室（北京市东城区天坛西里 4 号）
电话：010-57099318
Email：kq_yyll@163.com</td></tr>
</table>
