# Supplementary material for: Biomechanical analysis of the maxillary sinus floor membrane during internal sinus floor elevation with implants at different angles of the maxillary sinus angles
Source: Int J Implant Dent. 2024 Mar 12;10:11. doi: 10.1186/s40729-024-00530-5 (PMC10933249; doi:10.1186/s40729-024-00530-5)
Supplement: Supplementary file 3 — Supplementary Material 3 [file 40729_2024_530_MOESM3_ESM.docx]

**Title**

Biomechanical analysis of the maxillary sinus floor membrane during internal sinus floor elevation with implants at different angles of the maxillary sinus

**Author Contributions**

(I) Conception and design: Yinxin Deng; (II) Data analysis and interpretation: Yinxin Deng, Ruihong Ma, Yilin He; (III) Drafting article: All authors; (IV) Administrative support: Pan Ma; (V) Provision of study materials or patients: All authors; (VI) Final approval of manuscript: All authors.

**Availability of data and materials**

The datasets of the current study are available from the corresponding author on reasonable request.

**Fundings**

This work was supported by the National Natural Science Foundation of China (Grant number [81974153]), Beijing Municipal Natural Science Foundation (Grant number [L222088]) and the Innovation Research Team Project of Beijing Stomatological Hospital, Capital Medical University (Grant number [CXTD202204]).

**Acknowledgements**

The authors would like to thank American Journal Experts (www. china.aje.com) for English language editing.

**Authors details**

Yinxin Deng^1^, Ruihong Ma^2^, Yilin He^2^, Shujia Yu^2^, Shiyu Cao^2^, Kang Gao^2^, Yiping Dou^2^, Pan Ma^2^

^1^ Department of Stomatology, Beijing Hospital of Integrated Traditional Chinese and Western Medicine, Beijing, China

^2^ Dental Implant Center, Beijing Stomatological Hospital, School of Stomatology, Capital Medical University, Beijing, China

**Corresponding Author**

Pan Ma

Dental Implant Center

Beijing Stomatological Hospital, School of Stomatology, Capital Medical University

4 Tiantan Xili, Dongcheng District, Beijing 100050, China

Email: [mapanxw@163.com](mailto:mapanxw@163.com).

**Declarations**

**Ethics approval and consent to participate**

This study was approved by the Ethics Committee of Beijing Stomatological Hospital of Capital Medical University (CMUSH-IRB-KJ-PJ-2018-06).

**Consent for publication**

All the authors have approved the publication of this manuscript.

**Competing interests**

The authors declare that they have no known competing financial interests or personal relationships that could have appeared to influence the work reported in this paper.
