# Supplementary material for: Biomechanical analysis of the maxillary sinus floor membrane during internal sinus floor elevation with implants at different angles of the maxillary sinus angles
Source: Int J Implant Dent. 2024 Mar 12;10:11. doi: 10.1186/s40729-024-00530-5 (PMC10933249; doi:10.1186/s40729-024-00530-5)
Supplement: Supplementary file 5 — Supplementary Material 5 [file 40729_2024_530_MOESM5_ESM.docx]

Biomechanical analysis of the maxillary sinus floor **membrane** during **internal sinus floor elevation** with implants at different angles of the **maxillary sinus** angles

**Yinxin Deng^1^, Ruihong Ma^2^, Yilin He^2^, Shujia Yu^2^, Shiyu Cao^2^, Kang Gao^2^, Yiping Dou^2^, Pan Ma^2^**

**Author details**

**Affiliations**

^1^ Department of Stomatology, Beijing Hospital of Integrated Traditional Chinese and Western Medicine, Beijing, China

^2^ Dental Implant Center, Beijing Stomatological Hospital, School of Stomatology, Capital Medical University, Beijing, China

**Email addresses**

Yinxin Deng: 1018881917@qq.com

Ruihong Ma: mrh13120151622@163.com

Yilin He: 879803509@qq.com

Shujia Yu: 736896239@qq.com

Shiyu Cao: 978923037@qq.com

Kang Gao: gao.kang@ccmu.edu.cn

Yiping Dou: 763607768@qq.com

**Corresponding Author**

Pan Ma

Dental Implant Center

Beijing Stomatological Hospital, School of Stomatology, Capital Medical University

4 Tiantan Xili, Dongcheng District, Beijing 100050, China

Email: [mapanxw@163.com](mailto:mapanxw@163.com).

**Abstract**

**Objective:**This study analyzed and compared the biomechanical properties of maxillary sinus floor mucosa with implants at three different maxillary sinus angles during a modified internal sinus floor elevation procedure.

**Methods:**3D reconstruction of the implant, maxillary sinus bone, and membrane were performed. The maxillary sinus model was set at three different angles. Two internal maxillary sinus elevation models were established, and finite element analysis was used to simulate the modified maxillary sinus elevation process. The implant was elevated to 10 mm at three maxillary sinus angles when the maxillary sinus floor membrane was separated by 0 mm and 4 mm. The stress of the maxillary sinus floor membrane was analyzed and compared.

**Results:**When the maxillary sinus floor membrane was separated by 0 mm and elevated to 10 mm, the peak stress values of the implant on the maxillary sinus floor membrane at three different angles were as follows: maxillary sinus I: 5.14–78.32 MPa; maxillary sinus II: 2.81–73.89 MPa; and maxillary sinus III: 2.82–51.87 MPa. When the maxillary sinus floor membrane was separated by 4 mm and elevated to 10 mm, the corresponding values were as follows: maxillary sinus I: 0.50–7.25 MPa; maxillary sinus II: 0.81–16.55 MPa; and maxillary sinus III: 0.49–22.74 MPa.

**Conclusion:**The risk of sinus floor membrane rupture is greatly reduced after adequate dissection of the maxillary sinus floor membrane when performing modified internal sinus elevation in a narrow maxillary sinus. In a wide maxillary sinus, the risk of rupture or perforation of the wider maxillary sinus floor is reduced, regardless of whether traditional or modified internal sinus elevation is performed at the same height.

**KEYWORDS:** internal sinus elevation; finite element analysis; sinus floor membrane; maxillary sinus angle (anatomy)

**Introduction**

There is little alveolar bone available in the posterior maxillary region because of the presence of the maxillary sinus. The sparseness of bone in this region contributes to a relatively high rate of implant failure. Maxillary sinus elevation^1-3^ is necessary to address this problem and can involve either more typical external sinus elevation^4^ (lateral window maxillary sinus elevation via an opening in the anterior wall of the maxillary sinus) or less invasive internal sinus elevation^5,6^ (transcrestal maxillary sinus elevation via the alveolar crest). External maxillary sinus elevation^7^ involves stripping the membrane of the maxillary sinus floor under direct vision with high elevation. However, this procedure involves many regions and may cause additional trauma. The surgical procedure is complex and is associated with several postoperative side effects and a lengthy recovery period. In contrast, internal maxillary sinus elevation^8,9^ involves the use of a specialized osteotome to elevate the membrane of the maxillary sinus floor. It has the advantage of being less invasive, with a shorter operation time and fewer postoperative side effects. Many factors, including the residual alveolar bone height (RBH), maxillary sinus anatomy, and surgeon expertise, should be assessed when deciding on a preoperative surgical approach. Although there is still debate over this issue, the RBH has been identified as the main factor to be considered^10,11^. The current belief is that an internal maxillary sinus elevation procedure is relatively safe and reliable when the RBH is ≥ 4 mm. However, due to the highly sensitive nature of the technique, the application of internal maxillary sinus elevation is limited^12^ when the RBH is < 4 mm.

According to some researchers, internal maxillary sinus elevation is efficient and practicable^13-15^ when the RBH < 4 mm. In our clinical practice, we have also performed a modified internal sinus elevation procedure to facilitate internal sinus elevation even when the RBH < 4 mm. The modified internal sinus elevation procedure combines external maxillary sinus elevation with sinus mucoperiosteal stripping and internal sinus elevation through the top of the alveolar ridge. The membrane stripping procedure is performed on the maxillary sinus floor at the top of the alveolar ridge using a stripping instrument. This reduces the tension on the sinus mucoperiosteum, permitting greater elevation and simultaneous implant placement. We have successfully implemented the procedure in several cases. A retrospective clinical study of this modified procedure was performed, and the results^16^ have been successfully published in the Journal of Clinical Implant Dentistry and Related Research. However, in our subsequent use of the modified internal sinus elevation procedure, we have found that the difference in the angle of the maxillary sinus is an equally important factor.

Perforation of the maxillary sinus floor membrane is the most common complication^17-19^ of maxillary sinus floor elevation, regardless of the type of surgery^20,21^ performed or modifications^22-24^ made. Our retrospective analysis of clinical cases revealed that the structural makeup of the maxillary sinus (such as the membrane thickness, width, angularity, and septal condition) is an important but frequently disregarded aspect. The morphological classification of the maxillary floor wall and the rate of perforation in maxillary sinus elevation have been noted in many studies^25-28^. Based on cone-beam computed tomography (CBCT)^29,30^ imaging data of the maxillary sinus, other scholars have studied and categorized the maxillary sinus, primarily based on two aspects: the maxillary sinus angle^31^ and width^32^. Several scholars^25^ have noted that the risk of membrane perforation^27^ during maxillary sinus elevation is relatively increased in maxillary sinuses with small angles after combining imaging data and clinical cases. Others have shown that the width^33^ of the maxillary sinus determines the distance^34^ the sinus membrane can be stripped from the buccal wall to the palatal wall; the greater this distance is, the more difficult the stripping. Therefore, sufficient elevation of the membrane at the bottom of the sinus is difficult when the maxillary sinus is wider, resulting in a limited elevation height. Additionally, when the maxillary sinus is wide^35^, it is more challenging for the implant material to directly contact the buccal and palatal walls of the maxillary sinus. Furthermore, the sinus floor’s membrane also collapses downward due to excessive local tension after surgery, leading to resorption of the implant material. In essence, the width of the maxillary sinus and the proportion of new bone production are inversely correlated.

The morphology of the maxillary sinus is also directly related to the difficulty of performing procedures to elevate the maxillary sinus floor, the success rate, and the incidence of complications such as membrane perforation of the maxillary sinus floor. Preoperative imaging is essential for analyzing the morphology^36,37^ of the maxillary sinus and selecting the best surgical technique. When we perform the procedure of stripping the membrane at the floor of the sinus in clinical practice, we also find that the ease of stripping the sinus floor membrane and the height to which it can be elevated vary at different angles. Additionally, osteogenesis in the maxillary sinus differs among different angles after the modified internal maxillary sinus elevation procedure. We have found that in some patients with a flat maxillary sinus floor, after modified sinus elevation, although we successfully stripped the membrane of the sinus floor and placed the implant with bone grafting material, the postoperative osteogenesis was less than optimal. However, studies on whether the sinus floor membrane or elevation height is affected differently when implants are lifted at different maxillary sinus angles have yet to be reported.

Based on the analysis described above, a three-dimensional (3D) finite element model of the implant, bone and membrane in the maxillary sinus region was established during this experiment to dynamically simulate the separation and lifting of the maxillary sinus floor membrane and study the stress and strain of the maxillary sinus floor membrane when the implant is placed in the maxillary sinus model at different angles of internal sinus elevation. While this study focused on the stresses of the maxillary sinus floor membrane at different sinus angles, some of the 3D model images and experimental data were taken from a previous study by our team^16^. Rationality was achieved in vivo but not in vitro, utilizing a technique that is not invasive. The biomechanical studies will also offer a theoretical and practical foundation for the therapeutic application of modified internal sinus elevation.

**Materials and Methods**

1. **Experimental equipment**

Hardware: NewTom VG CBCT (Italy): voltage, 110 kV; current, 3.6 mA; reconstructed layer thickness, 0.125 mm.

Modeling software: Mimics 21.0 software (Materialise, Belgium).

Geomagic Studio2014 software (Raindrop, USA).

HyperMesh 14.0 software (Altair, USA).

Finite element analysis software: MSC Patran 2012 preprocessing software (NASA, USA).

MSC Nastran 2012 postprocessing software (NASA, USA).

1. **3D finite element modeling**
   1. **3D model reconstruction of the implant**

The ITI Straumann bone-level cylindrical implant, which is frequently used in clinical practice^38^ as a prototype, was used to establish an implant model, as shown in Figure 1, to study the different impacts of the implant tip on the membrane of the maxillary sinus floor during implant placement at various maxillary sinus angles during maxillary sinus elevation. The implant’s bottom end had a diameter of 4.8 mm, and its overall height was approximately 10.2 mm. The implant’s apical surface was rounded and had a diameter of 4.45 mm.


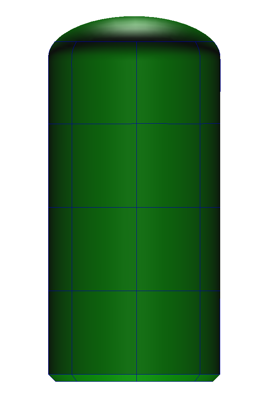


Figure 1 Reversed implant geometry model.

- 1. **3D model reconstruction of the maxillary sinus bone and membrane**

First, 3D model reconstruction was carried out using CBCT data in DICOM format.

As noted in the previous paragraphs, the maxillary sinus was classified in terms of angle and width (Figures 2 and 3)^36,37^. Additionally, we collected 80 maxillary sinus data points from patients who underwent modified internal sinus elevation (Figures 4–6) at the Oral Implantation Center of Beijing Stomatological Hospital, affiliated with Capital Medical University (Beijing, China) between February 2020 and July 2020. All patients were informed about the surgical plan and possible complications and provided signed informed consent forms, agreeing to further analysis of their CBCT data. The protocol for this study was designed following the World Medical Association Declaration of Helsinki for biomedical research involving human subjects. The local ethics committee approved the study (number CMUSH-IRB-KJ-PJ-2018-06).


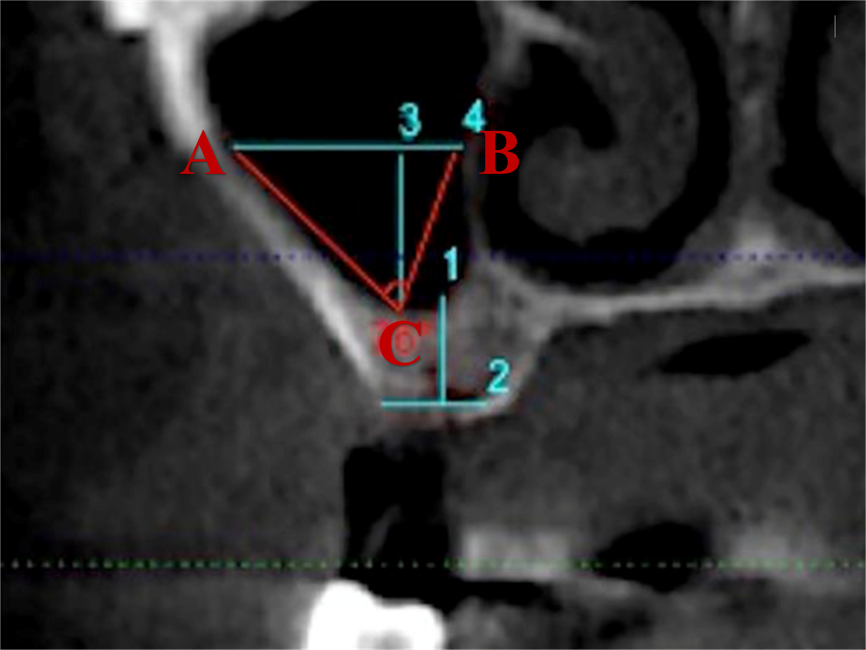


Figure 2 The investigated anatomical parameters^27^ measured on coronal preoperative CBCT. Maxillary sinus width (L_AB_): Horizontal distance between the buccal and palatal walls of the maxillary sinus at 10 mm above the lowest point of the maxillary sinus floor at the intended implantation site. Maxillary sinus angle (∠ ACB): Angle between a horizontal line drawn 10 mm above the lowest point of the maxillary sinus floor at the intended implantation site and the buccal wall bone plate and palatal wall bone plate of the maxillary sinus.


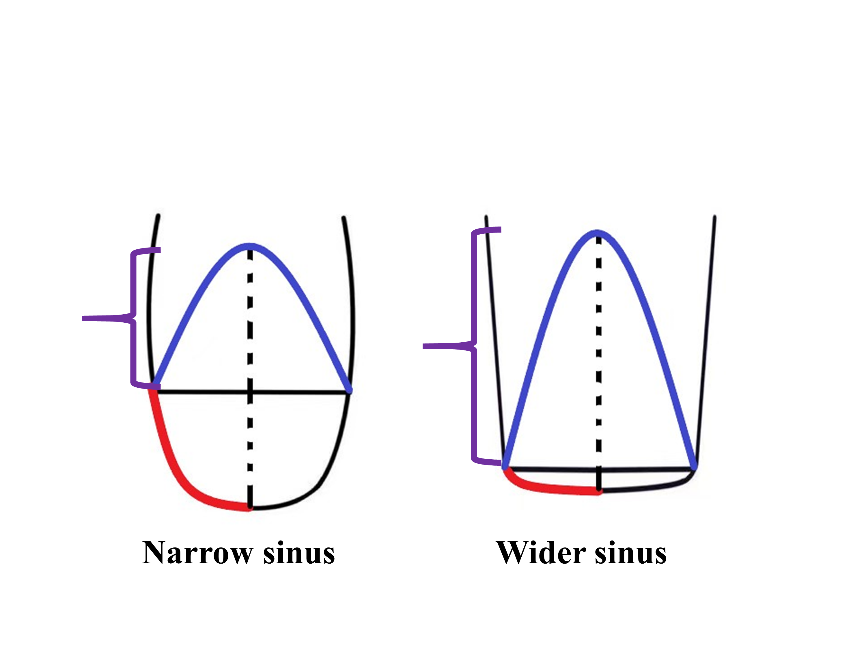


Figure 3 Schematic representation of the extent of membrane stripping and the height to which it can be elevated. The vertical distance to which the membrane is lifted by the tip of the implant (the height shown by the purple line and the portion of the membrane that is under tension shown by the blue line) is less in the narrower maxillary sinus (the red line shows the extent of the stripped mucosa) than in the wider maxillary sinus after membrane stripping.


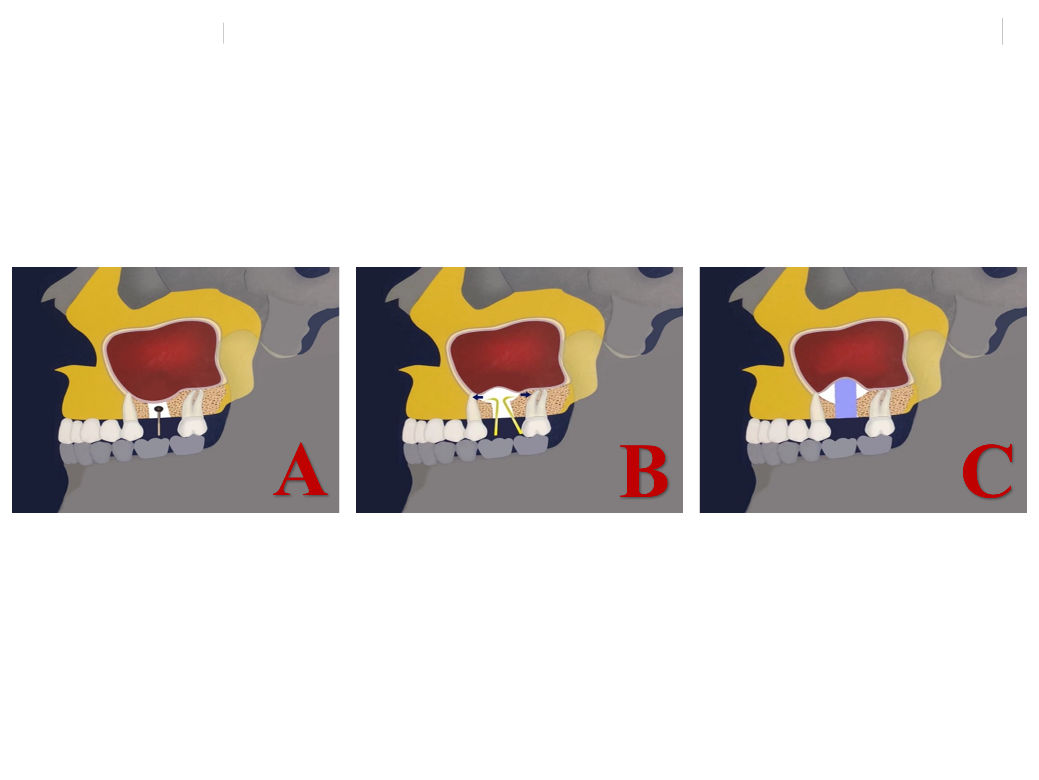


Figure 4 Illustrations of the modified internal sinus elevation procedure. (A) A Summer bone chisel was used to softly tap the alveolar crest, and this bone block was used as the roof of the area of elevated maxillary sinus floor. (B) Separation of the maxillary sinus floor membrane from the alveolar crest using a mucoperiosteal stripper. (C) Implant placement.


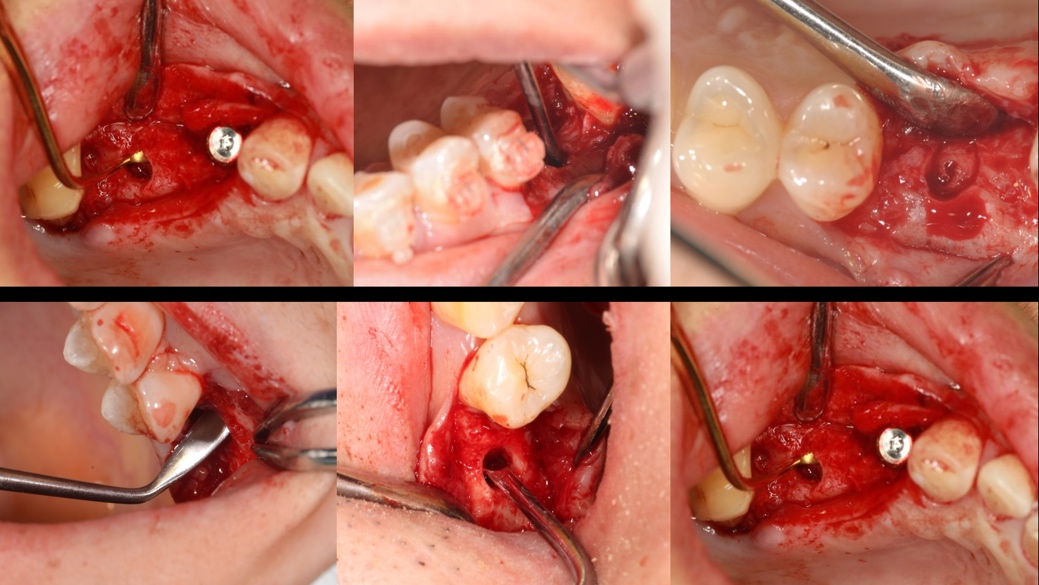


Figure 5 Separation of the maxillary sinus floor membrane/sinus mucoperiosteal detachment procedure.


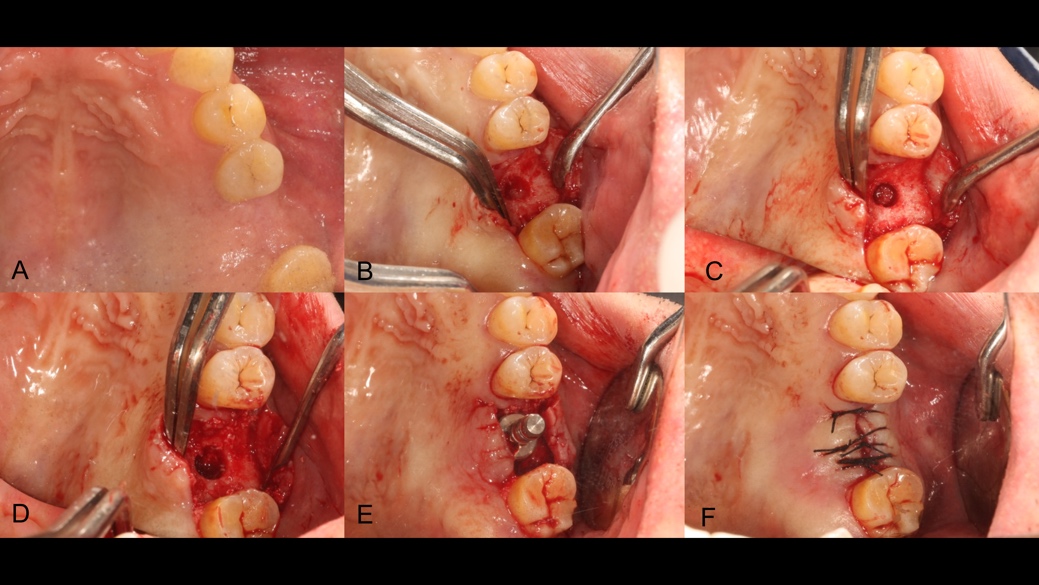

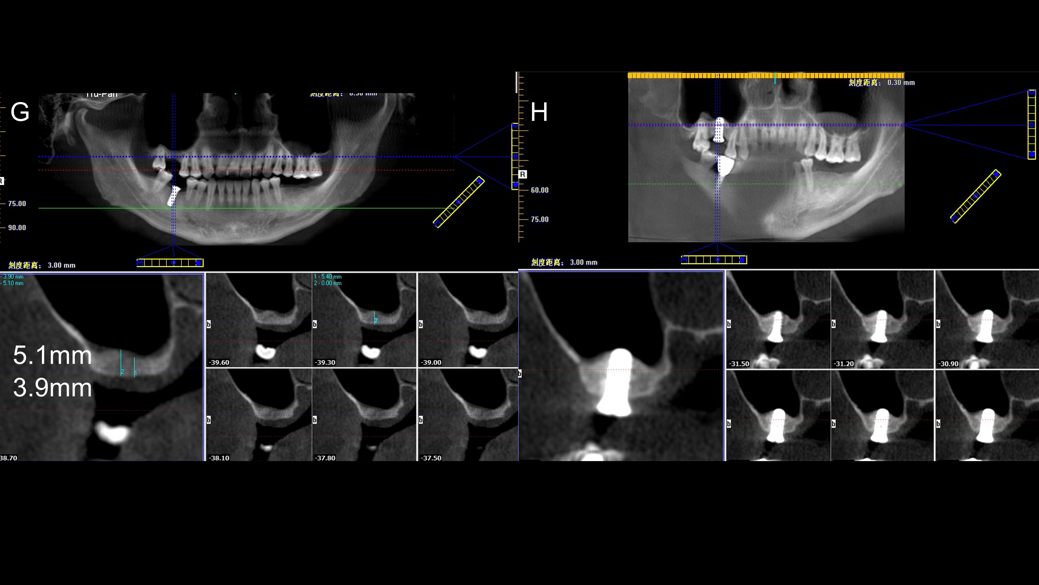


Figure 6 Surgical procedure for modified internal sinus elevation. (A) Preparation for surgery. (B) The alveolar crest is flat and slightly bluish. (C) Bone block serving as the roof of the area of elevated maxillary sinus floor. (D) Separation of the maxillary sinus floor membrane from the alveolar crest using a mucoperiosteal stripper. (E) Implant placement implant (the bone condensing technique was used to obtain good initial stability). (F) Tight suturing after implantation. (G) CBCT before surgery. (H) CBCT immediately after surgery.

The CBCT data were divided into groups based on the various maxillary sinus angles, as shown in Table 1.

Table 1. Maxillary sinus angle measurements

| Maxillary sinus angle | Number |
| --- | --- |
| <70° | 5 |
| 70°— 85° | 34 |
| 86°— 100° | 36 |
| >100° | 5 |

We chose the 85° maxillary sinus in the median group to represent the medium-width maxillary sinus. Moreover, we comparatively studied the stress distribution characteristics of the mucosa at the maxillary sinus floor between the narrower maxillary sinus and the wider maxillary sinus during the modified maxillary sinus elevation procedure. We also adjusted the angle of the maxillary sinus ourselves in Geomagic Studio 2014 software, creating 45° and 125° maxillary sinus models; to simplify the model, the two maxillary sinuses with their respective angles were constructed in a symmetrical bowl-shaped structure. After the modeling was finished, maxillary sinus grouping was performed as follows:

Maxillary sinus I: 45°, representing a narrow sinus.

Maxillary sinus II: 85°, representing a medium-width sinus.

Maxillary sinus III: 125°, representing a wide sinus.

We used Mimics 21.0 software for data extraction to reconstruct 3D geometric models of the maxillary sinus and mucosa and exported the file in STL format. Next, an overall processed geometric model of the maxillary sinus and mucosa was obtained in STP format in Geomagic Studio 2014 software by patching, noise reduction, and surfacing. Subsequently, the STP file was imported into HyperMesh 14.0 software for meshing, and the BDF file was then exported for finite element mesh property setting, material parameter definition, load application, boundary condition constraint, and various computational working condition analyses via the finite element preprocessing software MSC Patran 2012 and the finite element postprocessing software MSC Nastran 2012 for the analysis of different computational working conditions.

The process of manipulating the geometric model for 3D reconstruction of the maxillary sinus bone and membrane is shown in Figures 7 and 8.


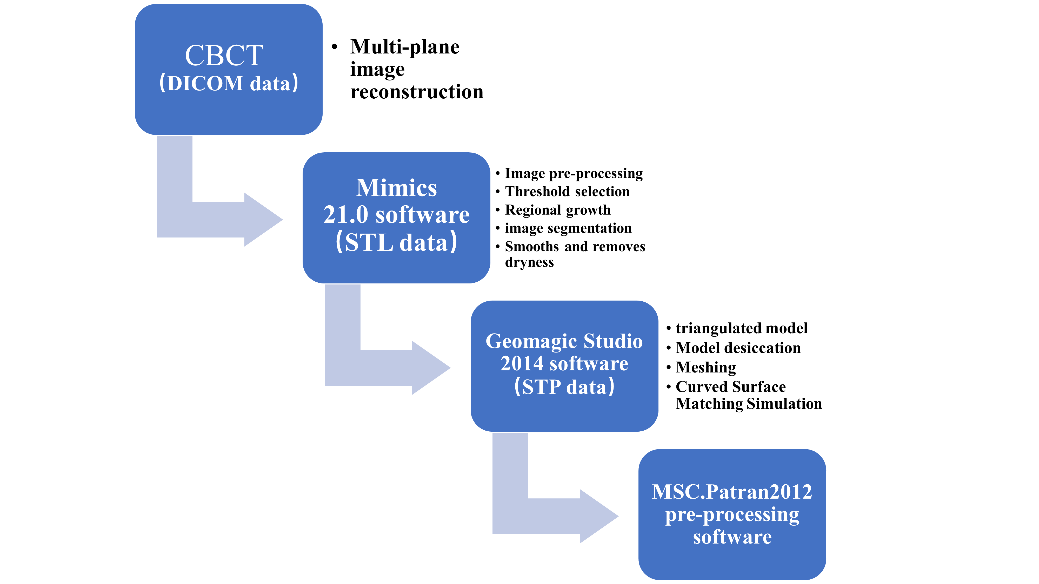


Figure 7 3D reconstruction model operation procedure.


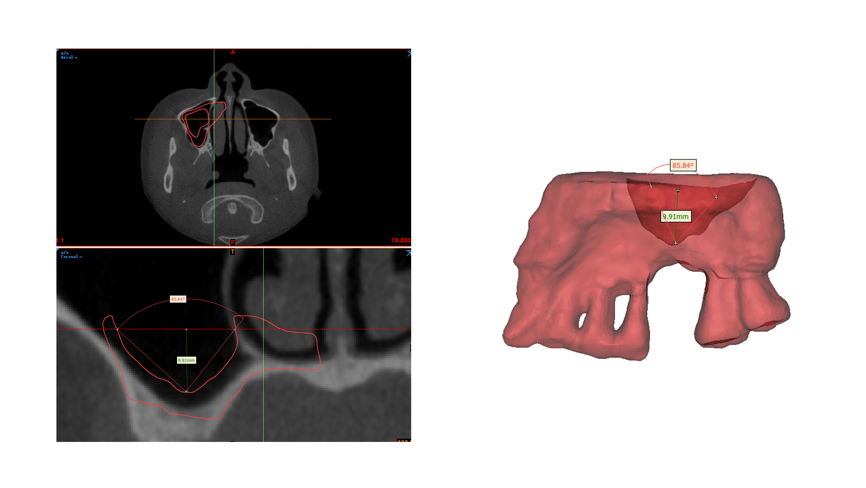

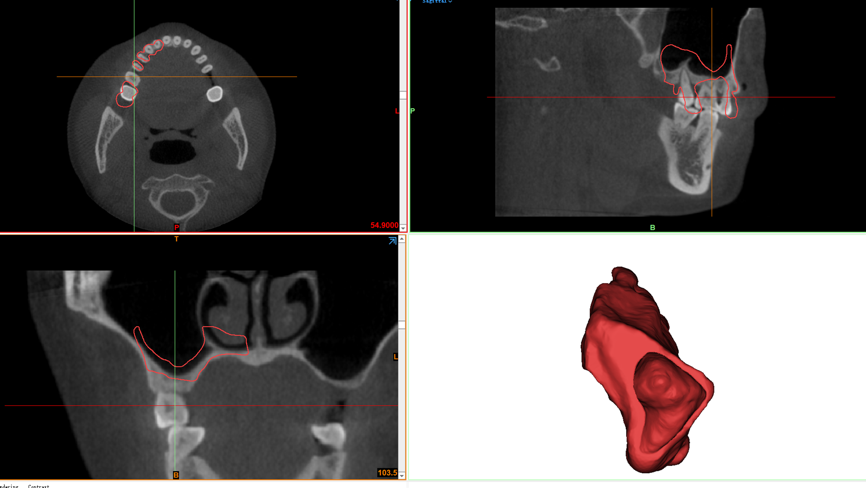


Figure 8 Schematic diagram of the 85° maxillary sinus as an example. Membrane CBCT data were extracted and then fitted to the bone model for matching and fitting.

The solid geometric models of the bone and membrane of the maxillary sinus at different angles were inverted and assembled with the geometric model of the implant, as shown in Figure 9.


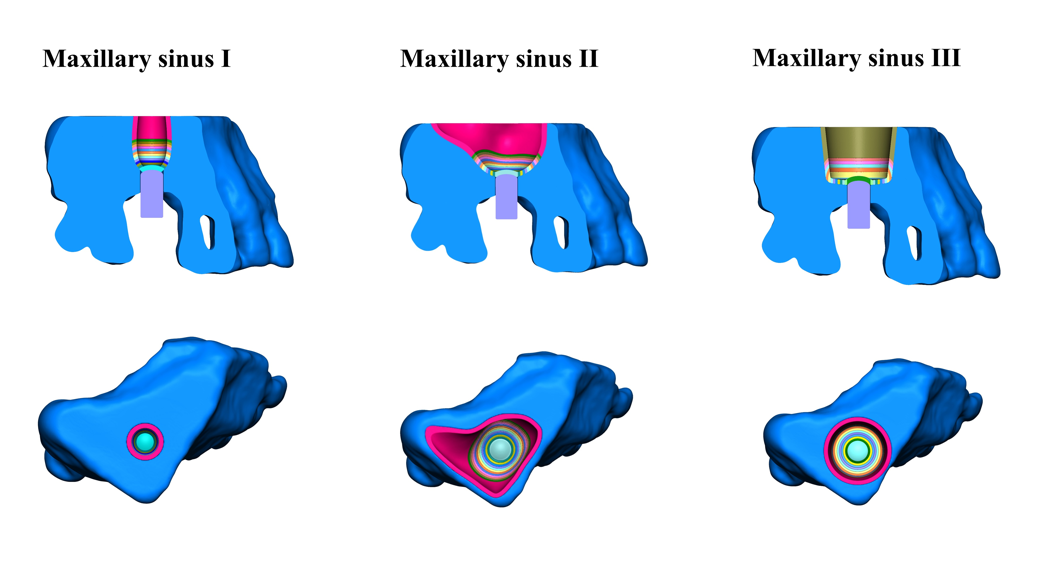


Figure 9 Reverse processing of solid geometric maxillary sinus bone and membrane models.

The geometric solid models corresponding to the three groups of maxillary sinus bones, membranes and implants were inverted and imported into MSC Patran 2012 software for structural assembly, as shown in Figure 10.


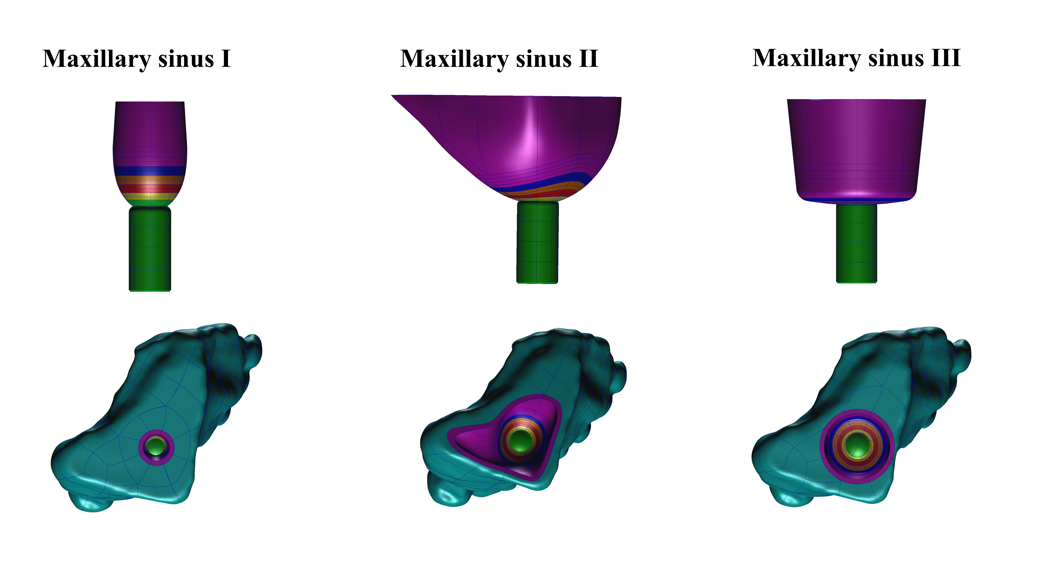


Figure 10 Geometric models imported into finite element preprocessing software.

**2.3 Finite element meshing**

The STP files of the implant and corresponding maxillary and sinus membrane models were imported into HyperMesh 14.0 software (Altair Engineering, Inc., Troy, MI, USA) for meshing. The finite element mesh property settings, material parameter definitions, loading conditions, and boundary condition constraints were applied in MSC Patran 2012 software, as shown in Figure 11.


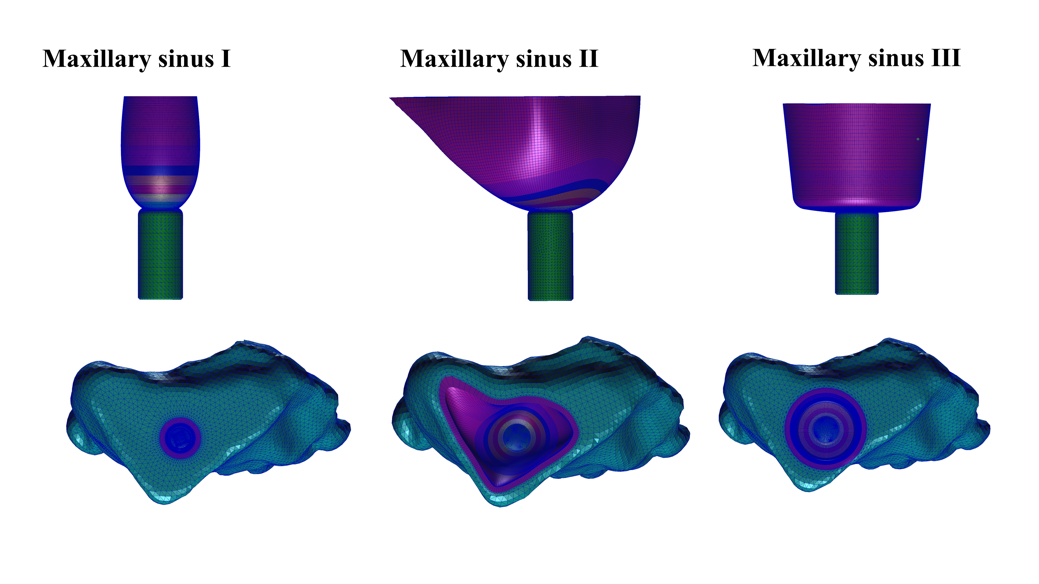


Figure 11 Finite element mesh maxillary sinus and membrane models imported into MSC Patran 2012.

The parameters used to mesh the finite element model of the maxillary sinus membrane at three different angles are shown in Table 2. The maxillary sinus bone, membrane, and implant were all meshed with solid units, and to improve the calculation accuracy, convergence, and efficiency, the membrane was divided into hexahedral mesh units (IsoMesh Hex8 Element), and the implant and maxilla were divided into tetrahedral mesh units (TetMesh Tet4 Element).

Table 2. Finite element model mesh parameters for maxillary sinus membrane at three different angles.

|  | Maxillary sinus I | Maxillary sinus II | Maxillary sinus III |
| --- | --- | --- | --- |
| Nodes | 295809 | 361514 | 590735 |
| Elements | 563688 | 367466 | 550882 |

**2.4 Biomechanical properties**

The mechanical properties of the material for each structure are shown in Table 3.

| Table 3. Mechanical properties^39^ of materials for the maxilla, sinus membrane, and implant structures | | |
| --- | --- | --- |
| Materials | Elastic modulus (MPa) | Poisson’s ratio |
| Cortical bone of the maxilla | 13700 | 0.30 |
| Cancellous bone of the maxilla | 1370 | 0.30 |
| Sinus membrane | Hyperelastic C_10_=0.253, C_01_=0.027 | |
| Implant (titanium) | 110000 | 0.35 |

The maxillary sinus membrane is a large-deformation nonlinear soft tissue with hyperelastic mechanical properties; thus, nonlinear, large-displacement deformation of the complex material is needed to allow the computation to be equivalent to the maxillary sinus membrane in terms of hyperelastic properties. The equivalence method is briefly described, as follows:

Considering the approximately isotropic, incompressible, hyperelastic material properties of the maxillary sinus membrane, which can be expressed analytically by the associated stress‒strain relationship, the measured data can be fitted to obtain the material parameters. A typical stress versus stretch form of a Mooney-Rivlin model for hyperelasticity was chosen for the curve fitting to evaluate the material parameters, where C10 and C01 are two typical material parameter coefficients for a hyperelastic Mooney–Rivlin model. Experimental data fitting was achieved via the Tools–Modeling–Experimental Data Fitting function in the MSC Patran/Nastran 2012 software. The measured stress‒strain curve data of the relevant materials were input through the Select Test Data command, and finally, the Mooney–Rivlin type was selected to calculate the properties for equivalent fitting; the process of equivalent fitting is shown in Figure 12.

**
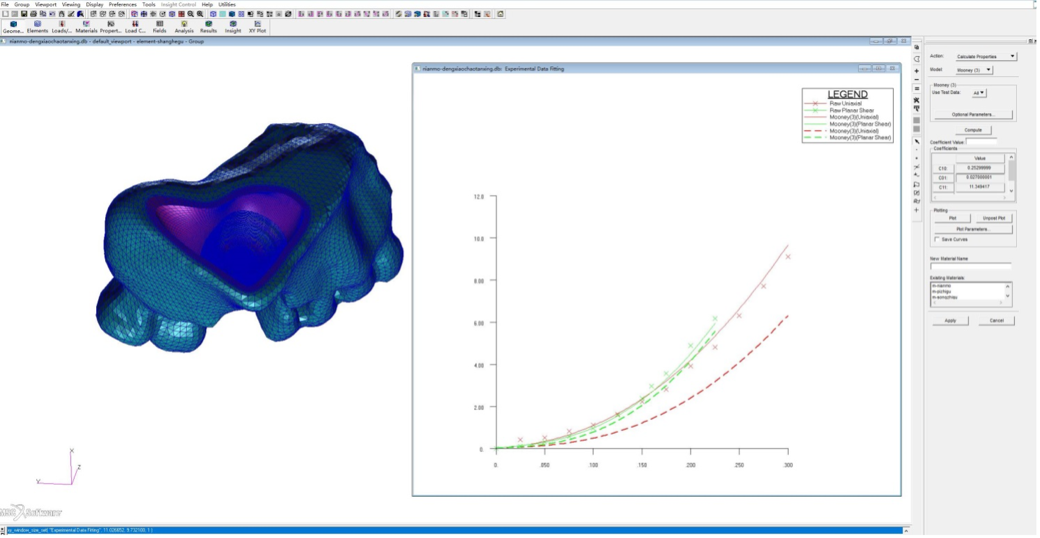
**

Figure 12 Hyperelastic material parametric equivalent fitting of the maxillary sinus membrane.

**3. Loading and constraint conditions**

The material assumptions, boundary limitations, and loading conditions of the structures were as follows:

- Nodes that constrained the surface of the maxilla were fixed, limiting their freedom of motion in three directions.
- The maxilla and implants were assumed to be isotropic, homogeneous, and continuous linear elastic materials, and the sinus membrane was equivalent to a hyperelastic material (nonlinearity, large displacement, and large deformation).
- During implant movement, only displacement in the direction of the pushing height was permitted; lateral displacement was restricted.
- When different degrees of membrane separation were simulated, the unseparated membrane region was considered the common node, while the membrane of the separated region was not in contact with the node of the corresponding maxillary region and was not considered the common node.
- The implant tip was considered to have frictional contact with the bottom of the membrane, the implant periphery was not in contact with the maxillary cavity preparation region, and the separated and nonseparated membranes were processed as a common node.

**4. Experimental model groupings**

The implants were elevated to different heights (1–10 mm) at three different maxillary sinus angles. The membrane of the maxillary sinus floor was also stripped by 0 mm and 4 mm, representing the traditional internal sinus elevation procedure and the modified internal sinus elevation procedure that we performed, respectively. The experimental model groupings are shown in Figure 13.


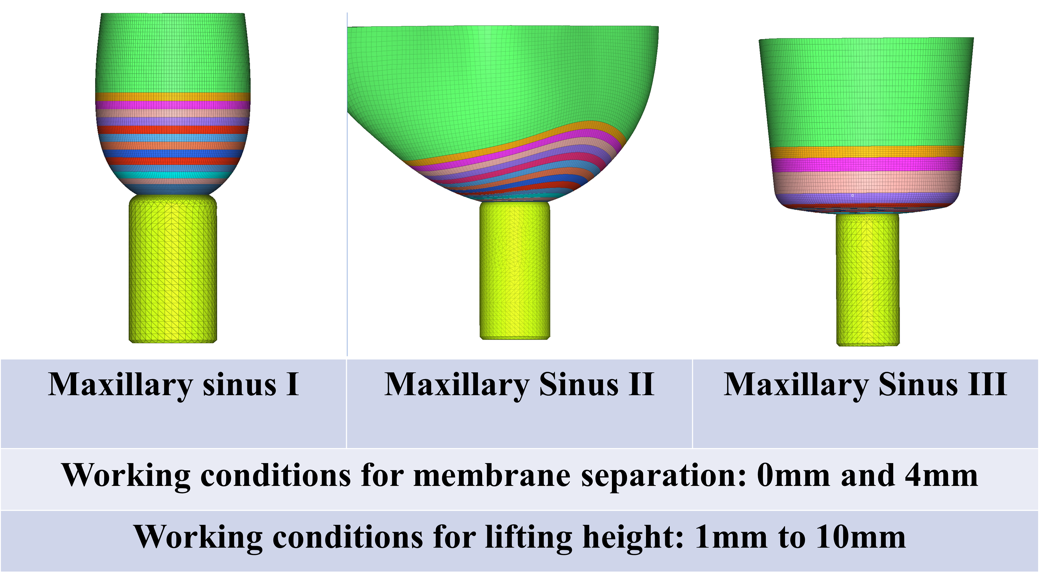


Figure 13 Experimental model groupings.

**5. Finite element analysis**

The finite element simulation analysis was performed on models under various working conditions before postprocessing in MSC Nastran 2012 software (NASA, USA), which was used for computational analysis and visualization of the results. The stress distribution nephogram and peak stress of the maxillary sinus membrane structure were obtained for each model group. The primary observation indicator was the value of the equivalent von Mises stress of the maxillary sinus floor membrane. The computational software is shown in Figure 14.


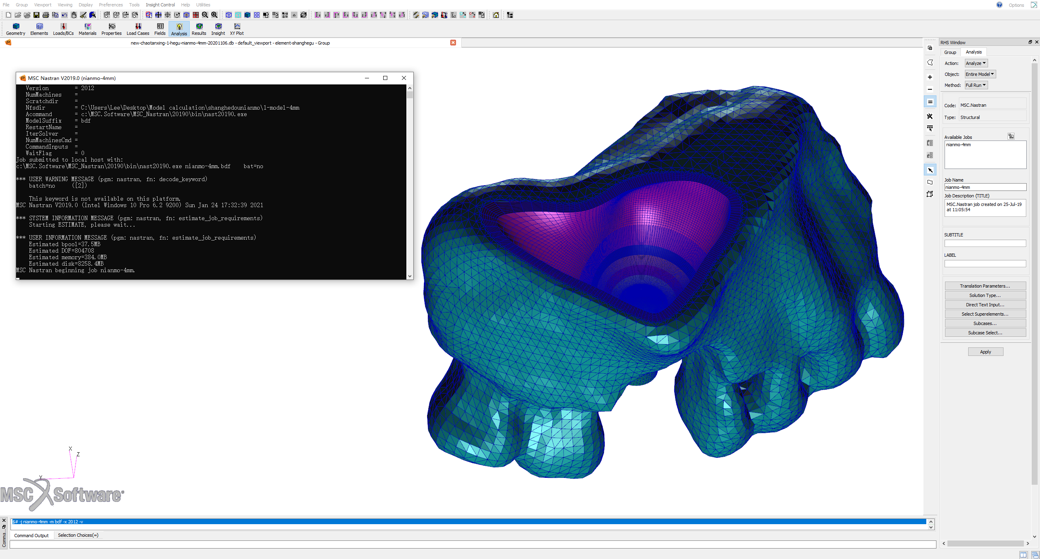


Figure 14 Finite element analysis computational software screen.

**Results**

For models under each working condition, the stress distribution nephogram of the maxillary sinus floor membrane was observed from the inferior view, the peak stress was calculated, the features of the stress distribution were examined, and graphs were created to compare the peak membrane stress values of each group of models.

The nephograms (Figure 15) illustrate the three different angles of the maxillary sinus floor membrane stress distribution with an elevation height of 10 mm and 0 mm and 4 mm degrees of sinus membrane separation.


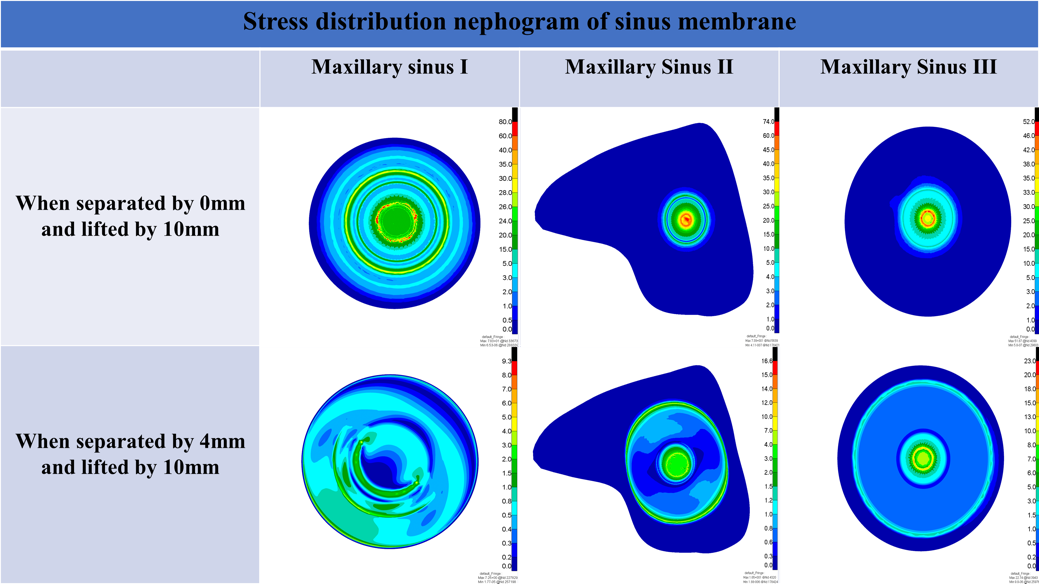


Figure 15 Stress distribution nephograms of the sinus membrane.

1. The peak membrane stresses (MPa) at three different maxillary sinus angles and 0 mm of maxillary sinus floor membrane separation are shown in Table 4 and Figure 16.

Table 4. Peak membrane stress (MPa) at 0 mm of separation for three different maxillary sinus angles.

| Elevation height  (mm) | Maxillary sinus I | Maxillary sinus II | Maxillary sinus III |
| --- | --- | --- | --- |
| 1 | 5.14 | 2.81 | 2.82 |
| 2 | 8.97 | 4.84 | 4.03 |
| 3 | 15.88 | 7.34 | 6.12 |
| 4 | 23.88 | 10.21 | 11.92 |
| 5 | 28.22 | 17.10 | 17.47 |
| 6 | 35.51 | 20.94 | 22.27 |
| 7 | 42.29 | 31.73 | 34.54 |
| 8 | 48.40 | 39.40 | 43.28 |
| 9 | 62.33 | 48.42 | 48.16 |
| 10 | 78.32 | 73.89 | 51.87 |


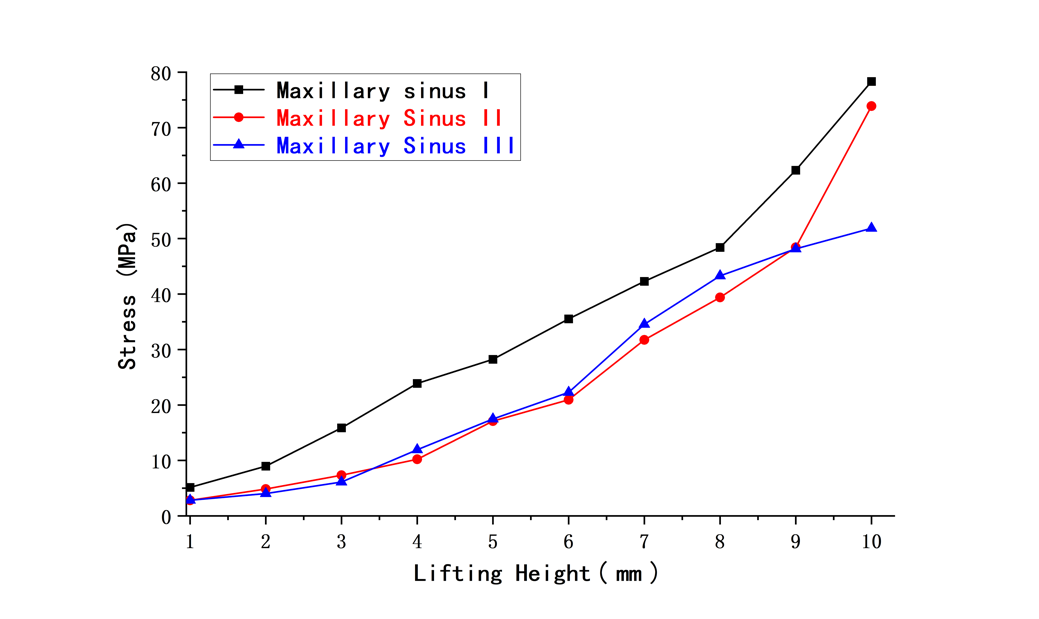


Figure 16 Peak maxillary sinus floor membrane stress at 0 mm of separation for three working conditions.

1. The peak membrane stresses (MPa) at three different maxillary sinus angles and 4 mm of maxillary sinus floor membrane separation are shown in Table 5 and Figure 17.

Table 5. Peak membrane stress (MPa) at 4 mm of separation for three different maxillary sinus angles.

| Elevation height  (mm) | Maxillary sinus I | Maxillary sinus II | Maxillary sinus III |
| --- | --- | --- | --- |
| 1 | 0.50 | 0.81 | 0.49 |
| 2 | 1.27 | 0.73 | 0.68 |
| 3 | 1.73 | 0.92 | 1.24 |
| 4 | 2.26 | 1.25 | 2.03 |
| 5 | 8.74 | 2.42 | 3.70 |
| 6 | 9.20 | 3.91 | 7.55 |
| 7 | 8.34 | 4.33 | 10.81 |
| 8 | 9.26 | 8.39 | 12.91 |
| 9 | 7.95 | 15.07 | 15.88 |
| 10 | 7.25 | 16.55 | 22.74 |


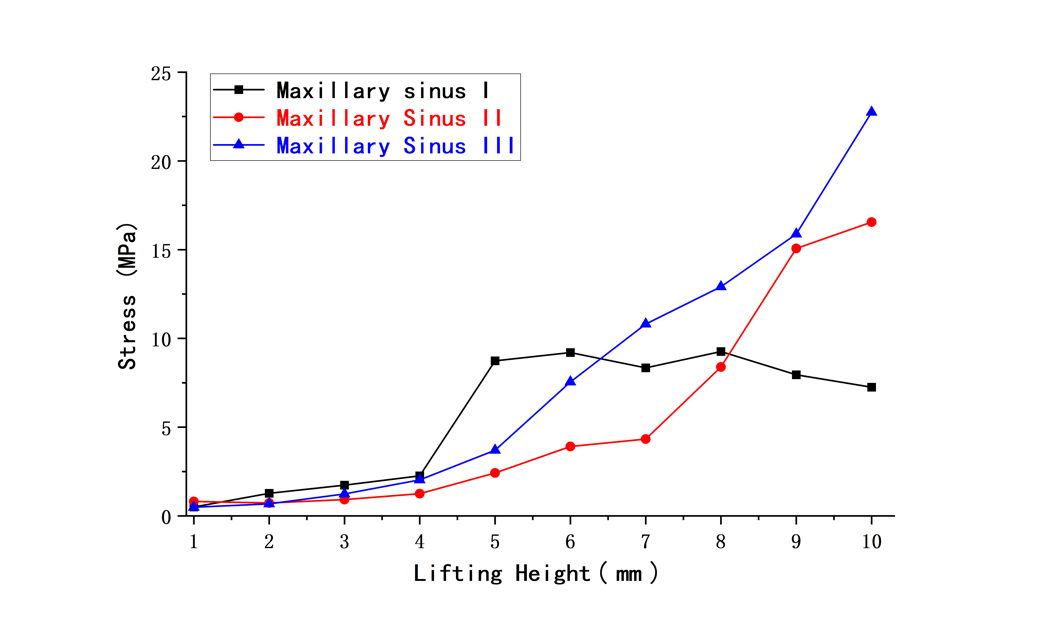


Figure 17 Peak maxillary sinus floor membrane stress at 4 mm of separation for three working conditions.

Figure 18 shows a comparison of the maxillary sinus stress at 0 mm and 4 mm of sinus floor membrane separation and three different maxillary sinus angles.


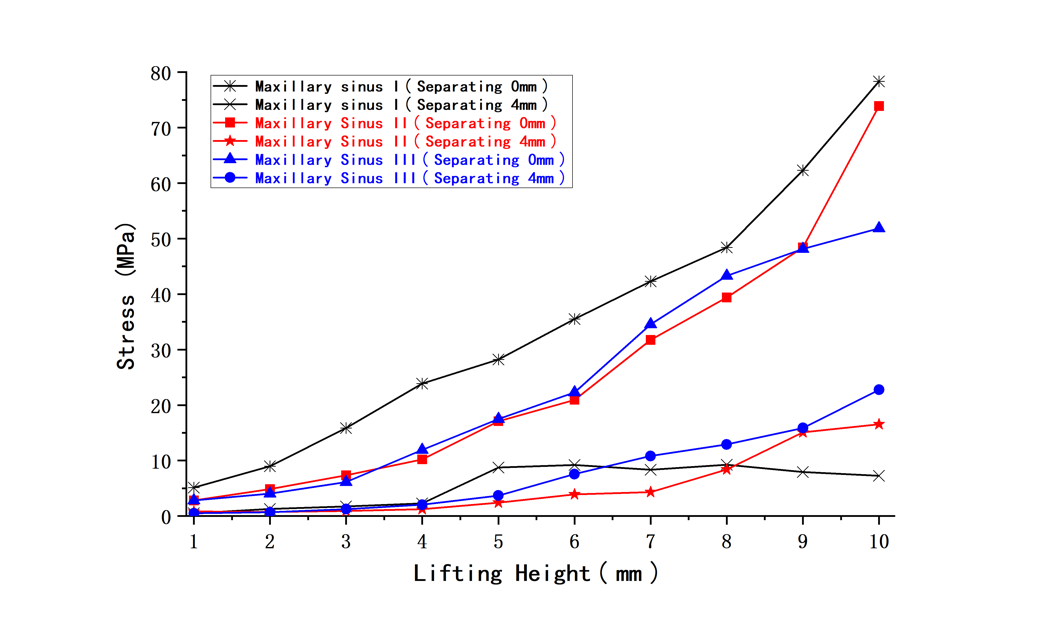


Figure 18 Peak maxillary sinus floor membrane stress at 0 mm and 4 mm of separation for three working conditions.

**Discussion**

Given the results of this finite element analysis, we were able to outline some useful recommendations regarding the preoperative planning of internal sinus elevation, the radiographic assessment and the sinus mucoperiosteal separation procedure.

1. **Experimental methodology and parameter design**

The finite element method^40^ regards the research object as a continuous elastic unit. This unit is decomposed into a series of minor finite object units, that is, finite elements, which then forms an aggregate of units to replace the original continuum and allow the overall stress distribution to be examined by studying the properties of each unit one by one. Therefore, the method can reflect the stresses, strains, and displacements in each part of the structure, especially the inner parts. The finite element method has progressed from simple 2D structural analysis to complex 3D structural analysis methods and has been widely applied in oral biomechanics^41,42^. Through in vitro experiments, we have learned about the mechanical and deformation characteristics of the maxillary sinus membrane^43,44^. Perforation of the membrane occurs only once the pressure applied during the peeling process exceeds the membrane's acceptance constraint, as determined in the previously mentioned in vitro experiments. The risk of perforation can be avoided only by limiting the height of elevation and precisely assessing anatomical locations because it is challenging to manage the force of punching and peeling during the actual procedure. Although there have been few published studies^39^ on the material constitutive and failure strength of the maxillary sinus membrane, determining the actual failure strength requires many cadaveric tests and relevant theoretical research. Therefore, we used the finite element analysis method to calculate the equivalent hyperelastic properties of the maxillary sinus mucosa through matching to obtain more similar hyperelastic C10 and C01 parameters; then, we input them into the finite element software for calculation and analysis from a qualitative point of view to study the force characteristics of the mucosa on the maxillary sinus.

The absolute values of the finite element calculation results are hardly representative of the actual values of the human body. However, if the clinical operation is correctly simulated, under the assumptions of the boundary conditions, simulated loading conditions, and impact loading conditions, the stresses and displacements generated at the interface show the same trend, and the results can play a specific role in guiding clinical operations. In this study, the method of impact loading was used, the calculations were smooth, and converged results were obtained. The trend of the stress and displacement values at the interface indicates that our model shows geometrically solid and biomechanical similarities to in vivo conditions and that the design of the experimental parameters is reasonable and can provide guidance for clinical operations. However, further research is still needed to create a computational model that completely replicates the intricate oral biomechanical environment.

**2. Modeling basis and geometric accuracy of the 3D finite element model**

To simulate internal sinus elevation as realistically as possible, we used the preoperative CBCT data of patients who had already undergone the modified internal sinus elevation procedure in the clinical setting for accurate modeling. Moreover, because of the diversity of maxillary morphology, in this study, we qualitatively compared only the stress, strain, and displacement of the maxillary sinus floor membrane during the rotational insertion of the implant. We also simplified the model to a certain extent by simulating only the bone in the maxillary sinus region instead of the intact maxilla, which did not affect the comparison of the results. In finite element analysis, models are commonly simplified by omitting secondary aspects for complex entities and making certain assumptions for test conditions.

The thickness of the maxillary sinus membrane exhibits considerable interindividual differences. Many researchers^45-47^ have measured and studied the thickness of the maxillary sinus membrane. Most scholars^48,49^ have used CBCT data in their work, while a few have measured mucosal tissue directly from cadaveric specimens. CBCT measurement errors can cause imaging measurements to be greater than those obtained from cadaveric specimens. Ongoing discussion exists on a standard imaging threshold for the maxillary sinus mucosal thickness. With most studies using 1 mm as a standard^50,51^, we simplified the maxillary sinus membrane by setting its thickness to 1 mm.

While lifting the maxillary sinus membrane with internal sinus elevation, we concentrated on maxillary sinus floor membrane perforation. To investigate and compare the test results under different working conditions, we focused on the maxillary sinus membrane under the assumption of boundary conditions after assembling and meshing the geometric model. This test also allowed the analysis of changes in the biomechanical properties of the maxillary sinus membrane with different degrees of separation from a qualitative standpoint. This calculation and analysis may also support the accuracy of our modified internal sinus elevation model.

**3. Comparison of peak stresses in the membrane of the maxillary sinus floor under different working conditions**

The above experimental results revealed that at 0 mm of maxillary sinus floor membrane separation, with increasing elevation, the maxillary sinus floor membrane in the three corresponding maxillary sinus angle groups showed a nonlinear increase in force. However, the magnitude and amplitude of the increase varied. At different internal elevation heights, the peak membrane stress of maxillary sinuses II and III was notably lower than that of maxillary sinus I, with decreases ranging from approximately -57.2% to -5.7% and -61.5% to -10.6%, respectively. For both maxillary sinuses II and III, the stress of the maxillary sinus floor membrane at 4 mm of separation gradually increased with increasing elevation, whereas the corresponding stress of maxillary sinus I showed an increasing and then slowly decreasing trend. At an elevation height of 6 mm or less, the peak membrane stress of maxillary sinuses II and III was lower than that of maxillary sinus I, with decreases of approximately -72.3% to -9.4% and -57.7% to -2.0%, respectively. At an elevation height of 6.5 mm or more, the peak membrane stress of maxillary sinus III was greater than that of maxillary sinuses I and II. The increase in membrane stress compared to that in maxillary sinus I was approximately 29.6% to 213.7%, while the increase in membrane stress in maxillary sinus II compared to that in maxillary sinus I at an elevation height of 8 mm or more was approximately 89.6% to 128.3%. These characteristic changes in stress may be related to the morphological changes in the mucosal structure caused when separating the mucosa of the floor of the narrower maxillary sinus.

We discovered variations in the efficiency of maxillary sinus floor elevation with different angles and morphologies, in addition to the effects of the different morphologies (shallow concave, deep concave, and convex) on the stress and displacement of the sinus floor membrane that other scholars^52^ have previously studied. Here, elevation efficiency can be understood as the amount of stress on the mucosa of the maxillary sinus floor during elevation, with less stress when elevating to the same height indicating greater efficiency. At 0 mm of separation, a deep concave sinus floor approximately corresponds to maxillary sinuses II and III defined in this study, and a shallow concave sinus floor approximately corresponds to maxillary sinus I. These results show that at the same elevation height, the peak mucosal stress on the floor of both maxillary sinuses II and III is less than that of maxillary sinus I. We did not specifically model a convex sinus floor. However, in conjunction with our previous analyses, the present results suggest that the morphology of the sinus floor of maxillary sinus I is similar to that of a convex sinus floor with 4 mm of membrane separation and an elevation of more than 6 mm. When exposed to the impact, the convex maxillary sinus floor shows a more substantial stress dispersion effect. This also explains why the peak membrane stress of maxillary sinus I decreases beyond an elevation height of 6 mm at 4 mm of separation.

The peak membrane stress on the sinus floor at 4 mm of separation was lower than that at 0 mm for the same maxillary sinus under various operating conditions. Additionally, the nephogram indicates that the stress concentration quickly increases in the region where the implant tip is in contact with the membrane and intensifies with increasing elevation. The stress concentration phenomenon improved when the membrane was at 4 mm of separation. Therefore, sufficient decortication of the maxillary sinus membrane will improve the stress concentration and reduce the risk of rupture. This means that stripping the sinus floor membrane is straightforward and effective, and our modification is reliable and relatively safe.

**Conclusions**

With traditional internal sinus elevation, the peak membrane stress of maxillary sinus I was notably greater than that of maxillary sinuses II and III at different elevation heights. This means that the risk of rupture and perforation of the membrane at the floor of the narrow maxillary sinus was relatively high without separation of the floor membrane. In the modified internal sinus elevation procedure, the membrane stress of maxillary sinuses II and III tended to increase as the elevation height increased, whereas the stress of maxillary sinus I tended to increase and then slowly decrease. In other words, after separating the membrane of the sinus floor, the risk of rupture and perforation of the membrane of the narrow maxillary sinus was relatively low with greater elevation. In summary, the risk of maxillary sinus floor membrane rupture is greatly reduced after adequate stripping of the sinus floor membrane when performing modified internal sinus elevation in a narrow maxillary sinus. In wider maxillary sinuses, the risk of membrane rupture and perforation at the same elevation is relatively low, regardless of whether traditional or modified internal sinus elevation is performed.

**Abbreviations**

RBH: residual bone height; 3D: three-dimensional; CBCT: cone-beam computed tomography; STL: stereolithography; BDF: bitmap distribution format.

**Declarations**

**Ethical approval and consent to participate**

This study was approved by the Ethics Committee of Beijing Stomatological Hospital of Capital Medical University (CMUSH-IRB-KJ-PJ-2018-06).

**Consent for publication**

All the authors have approved the publication of this manuscript.

**Availability of supporting data**

The datasets used in the present study are available from the corresponding author upon reasonable request.

**Competing interests**

The authors declare that they have no known competing financial interests or personal relationships that could have appeared to influence the work reported in this paper.

**Authors' contributions**

(I) Conception and design: Yinxin Deng; (II) data analysis and interpretation: Yinxin Deng, Ruihong Ma, Yilin He; (III) article drafting: all authors; (IV) administrative support: Pan Ma; (V) provision of study materials or patients: all authors; (VI) final approval of manuscript: all authors.

**Funding**

This work was supported by the National Natural Science Foundation of China (Grant number 81974153), the Beijing Municipal Natural Science Foundation (Grant number L222088) and the Innovation Research Team Project of Beijing Stomatological Hospital, Capital Medical University (Grant number CXTD202204).

**References**

1. Boyne PJ, James RA. Grafting of the maxillary sinus floor with autogenous marrow and bone. *J Oral Surg*. 1980;38(8):613-6

2. Tatum HJ. Maxillary and sinus implant reconstructions. *Dent Clin North Am*. 1986;30(2):207-29

3. Summers RB. The osteotome technique: Part 3--Less invasive methods of elevating the sinus floor. *Compendium*. 1994;15(6):698, 700, 702-4 passim; quiz 710

4. Dongo V, von Krockow N, Martins-Filho P, Weigl P. Lateral sinus floor elevation without grafting materials. Individual- and aggregate-data meta-analysis. *J Craniomaxillofac Surg*. 2018;46(9):1616-1624

5. Pjetursson BE, Rast C, Bragger U et al. Maxillary sinus floor elevation using the (transalveolar) osteotome technique with or without grafting material. Part I: Implant survival and patients' perception. *Clin Oral Implants Res*. 2009;20(7):667-76

6. Bruschi GB, Crespi R, Cappare P, Gherlone E. Transcrestal sinus floor elevation: a retrospective study of 46 patients up to 16 years. *Clin Implant Dent Relat Res*. 2012;14(5):759-67

7. Wen Y, Wei D, Jiang X et al. Lateral sinus floor elevation in patients with sinus floor defects: A retrospective study with a 1- to 9-year follow-up. *Clin Oral Implants Res*. 2023;

8. Andres-Garcia R, Rios-Santos JV, Herrero-Climent M et al. Sinus Floor Elevation via an Osteotome Technique without Biomaterials. *Int J Environ Res Public Health*. 2021;18(3)

9. Lin ZZ, Jiao YQ, Ye ZY, Wang GG, Ding X. The survival rate of transcrestal sinus floor elevation combined with short implants: a systematic review and meta-analysis of observational studies. *Int J Implant Dent*. 2021;7(1):41

10. Urban IA, Lozada JL. A prospective study of implants placed in augmented sinuses with minimal and moderate residual crestal bone: results after 1 to 5 years. *Int J Oral Maxillofac Implants*. 2010;25(6):1203-12

11. Farina R, Riccardi O, Schincaglia GP et al. Six-year extension results of a randomized trial comparing transcrestal and lateral sinus floor elevation at sites with 3-6 mm of residual bone. *Clin Oral Implants Res*. 2023;34(8):813-821

12. Stacchi C, Bernardello F, Spinato S et al. Intraoperative complications and early implant failure after transcrestal sinus floor elevation with residual bone height </=5 mm: A retrospective multicenter study. *Clin Oral Implants Res*. 2022;33(8):783-791

13. Gonzalez S, Tuan MC, Ahn KM, Nowzari H. Crestal approach for maxillary sinus augmentation in patients with </= 4 mm of residual alveolar bone. *Clin Implant Dent Relat Res*. 2014;16(6):827-35

14. Bernardello F, Righi D, Cosci F et al. Crestal sinus lift with sequential drills and simultaneous implant placement in sites with <5 mm of native bone: a multicenter retrospective study. *Implant Dent*. 2011;20(6):439-44

15. Thomas TJ, Bidra AS. Internal Sinus Membrane Elevation in Patients With Less Than 5 mm Residual Bone Height. *Compend Contin Educ Dent*. 2018;39(5):e13-e16

16. Deng Y, Tong C, Gao K et al. Modified internal sinus elevation for patients with low residual bone height: A retrospective clinical study. *Clin Implant Dent Relat Res*. 2023;25(3):458-472

17. Hsu YT, Rosen PS, Choksi K et al. Complications of sinus floor elevation procedure and management strategies: A systematic review. *Clin Implant Dent Relat Res*. 2022;24(6):740-765

18. Nolan PJ, Freeman K, Kraut RA. Correlation between Schneiderian membrane perforation and sinus lift graft outcome: a retrospective evaluation of 359 augmented sinus. *J Oral Maxillofac Surg*. 2014;72(1):47-52

19. Gargallo-Albiol J, Tattan M, Sinjab KH, Chan HL, Wang HL. Schneiderian membrane perforation via transcrestal sinus floor elevation: A randomized ex vivo study with endoscopic validation. *Clin Oral Implants Res*. 2019;30(1):11-19

20. Diaz-Olivares LA, Cortes-Breton BJ, Martinez-Rodriguez N et al. Management of Schneiderian membrane perforations during maxillary sinus floor augmentation with lateral approach in relation to subsequent implant survival rates: a systematic review and meta-analysis. *Int J Implant Dent*. 2021;7(1):91

21. Tavelli L, Borgonovo AE, Saleh MH et al. Classification of Sinus Membrane Perforations Occurring During Transcrestal Sinus Floor Elevation and Related Treatment. *Int J Periodontics Restorative Dent*. 2020;40(1):111-118

22. Chen L, Cha J. An 8-year retrospective study: 1,100 patients receiving 1,557 implants using the minimally invasive hydraulic sinus condensing technique. *J Periodontol*. 2005;76(3):482-91

23. Kfir E, Kfir V, Eliav E, Kaluski E. Minimally invasive antral membrane balloon elevation: report of 36 procedures. *J Periodontol*. 2007;78(10):2032-5

24. Sohn DS, Lee JS, An KM, Choi BJ. Piezoelectric internal sinus elevation (PISE) technique: a new method for internal sinus elevation. *Implant Dent*. 2009;18(6):458-63

25. Danesh-Sani SA, Loomer PM, Wallace SS. A comprehensive clinical review of maxillary sinus floor elevation: anatomy, techniques, biomaterials and complications. *Br J Oral Maxillofac Surg*. 2016;54(7):724-30

26. Shrestha B, Shrestha R, Lin T et al. Evaluation of maxillary sinus volume in different craniofacial patterns: a CBCT study. *Oral Radiol*. 2021;37(4):647-652

27. Zheng X, Huang L, Huang S, Mo A, Zhu J. Influence of anatomical factors related to maxillary sinus on outcomes of transcrestal sinus floor elevation. *J Dent Sci*. 2022;17(1):438-443

28. Guncu GN, Yildirim YD, Wang HL, Tozum TF. Location of posterior superior alveolar artery and evaluation of maxillary sinus anatomy with computerized tomography: a clinical study. *Clin Oral Implants Res*. 2011;22(10):1164-1167

29. Bornstein MM, Seiffert C, Maestre-Ferrin L et al. An Analysis of Frequency, Morphology, and Locations of Maxillary Sinus Septa Using Cone Beam Computed Tomography. *Int J Oral Maxillofac Implants*. 2016;31(2):280-7

30. Neugebauer J, Ritter L, Mischkowski RA et al. Evaluation of maxillary sinus anatomy by cone-beam CT prior to sinus floor elevation. *Int J Oral Maxillofac Implants*. 2010;25(2):258-65

31. Craiu C, Rusu MC, Hostiuc S, Sandulescu M, Derjac-Arama AI. Anatomic variation in the pterygopalatine angle of the maxillary sinus and the maxillary bulla. *Anat Sci Int*. 2017;92(1):98-106

32. Bertl K, Mick R, Heimel P et al. Variation in bucco-palatal maxillary sinus width does not permit a meaningful sinus classification. *Clin Oral Implants Res*. 2018;29(12):1220-1229

33. Chan HL, Suarez F, Monje A, Benavides E, Wang HL. Evaluation of maxillary sinus width on cone-beam computed tomography for sinus augmentation and new sinus classification based on sinus width. *Clin Oral Implants Res*. 2014;25(6):647-52

34. Jang HY, Kim HC, Lee SC, Lee JY. Choice of graft material in relation to maxillary sinus width in internal sinus floor augmentation. *J Oral Maxillofac Surg*. 2010;68(8):1859-68

35. Niu L, Wang J, Yu H, Qiu L. New classification of maxillary sinus contours and its relation to sinus floor elevation surgery. *Clin Implant Dent Relat Res*. 2018;20(4):493-500

36. Pommer B, Ulm C, Lorenzoni M et al. Prevalence, location and morphology of maxillary sinus septa: systematic review and meta-analysis. *J Clin Periodontol*. 2012;39(8):769-73

37. Lozano-Carrascal N, Salomo-Coll O, Gehrke SA et al. Radiological evaluation of maxillary sinus anatomy: A cross-sectional study of 300 patients. *Ann Anat*. 2017;214:1-8

38. Fugazzotto PA, Vlassis J, Butler B. ITI implant use in private practice: clinical results with 5,526 implants followed up to 72+ months in function. *Int J Oral Maxillofac Implants*. 2004;19(3):408-12

39. Zhai M, Cheng H, Yuan J et al. Nonlinear Biomechanical Characteristics of the Schneiderian Membrane: Experimental Study and Numerical Modeling. *Biomed Res Int*. 2018;2018:2829163

40. Velloso GR, Vidigal GJ, de Freitas MM et al. Tridimensional analysis of maxillary sinus anatomy related to sinus lift procedure. *Implant Dent*. 2006;15(2):192-6

41. Abdulghani EA, Al-Sosowa AA, Alhammadi MS et al. Three-dimensional assessment of the favorability of maxillary posterior teeth intrusion in different skeletal classes limited by the vertical relationship with the maxillary sinus floor. *Head Face Med*. 2022;18(1):13

42. Koca OL, Eskitascioglu G, Usumez A. Three-dimensional finite-element analysis of functional stresses in different bone locations produced by implants placed in the maxillary posterior region of the sinus floor. *J Prosthet Dent*. 2005;93(1):38-44

43. Insua A, Monje-Gil F, Garcia-Caballero L et al. Mechanical characteristics of the maxillary sinus Schneiderian membrane ex vivo. *Clin Oral Investig*. 2018;22(3):1139-1145

44. Pommer B, Unger E, Suto D, Hack N, Watzek G. Mechanical properties of the Schneiderian membrane in vitro. *Clin Oral Implants Res*. 2009;20(6):633-7

45. Janner SF, Caversaccio MD, Dubach P et al. Characteristics and dimensions of the Schneiderian membrane: a radiographic analysis using cone beam computed tomography in patients referred for dental implant surgery in the posterior maxilla. *Clin Oral Implants Res*. 2011;22(12):1446-53

46. Guo ZZ, Liu Y, Qin L et al. Longitudinal response of membrane thickness and ostium patency following sinus floor elevation: a prospective cohort study. *Clin Oral Implants Res*. 2016;27(6):724-9

47. Lin YH, Yang YC, Wen SC, Wang HL. The influence of sinus membrane thickness upon membrane perforation during lateral window sinus augmentation. *Clin Oral Implants Res*. 2016;27(5):612-7

48. Makary C, Rebaudi A, Menhall A, Naaman N. Changes in Sinus Membrane Thickness After Lateral Sinus Floor Elevation: A Radiographic Study. *Int J Oral Maxillofac Implants*. 2016;31(2):331-7

49. Pommer B, Dvorak G, Jesch P et al. Effect of maxillary sinus floor augmentation on sinus membrane thickness in computed tomography. *J Periodontol*. 2012;83(5):551-6

50. Munakata M, Yamaguchi K, Sato D, Yajima N, Tachikawa N. Factors influencing the sinus membrane thickness in edentulous regions: a cone-beam computed tomography study. *Int J Implant Dent*. 2021;7(1):16

51. Kalyvas D, Kapsalas A, Paikou S, Tsiklakis K. Thickness of the Schneiderian membrane and its correlation with anatomical structures and demographic parameters using CBCT tomography: a retrospective study. *Int J Implant Dent*. 2018;4(1):32

52. Xu YZ, Zhang FQ, Xu Q, Yu JH, Wang RR, Shang GW. (2008). Effect of different configurations of maxillary sinus floor on the stress and displacement of bone condenser, bone and mucosa of maxillary sinus during internal sinus floor elevation: Three-dimensional finite element analysis. Journal of Clinical Rehabilitative Tissue Engineering Research;12(17):3252-3256(China)
