# Supplementary material for: Biomechanical analysis of the maxillary sinus floor membrane during internal sinus floor elevation with implants at different angles of the maxillary sinus angles
Source: Int J Implant Dent. 2024 Mar 12;10:11. doi: 10.1186/s40729-024-00530-5 (PMC10933249; doi:10.1186/s40729-024-00530-5)
Supplement: Supplementary file 7 — Supplementary Material 7 [file 40729_2024_530_MOESM7_ESM.docx]

Figure 1 Reversed implant geometry model.

Figure 2 The investigated anatomical parameters^27^ measured on coronal preoperative CBCT. Maxillary sinus width (L_AB_): Horizontal distance between the buccal and palatal walls of the maxillary sinus at 10 mm above the lowest point of the maxillary sinus floor at the intended implantation site. Maxillary sinus angle (∠ ACB): Angle between a horizontal line drawn 10 mm above the lowest point of the maxillary sinus floor at the intended implantation site and the buccal wall bone plate and palatal wall bone plate of the maxillary sinus.

Figure 3 Schematic representation of the extent of membrane stripping and the height to which it can be elevated. The vertical distance to which the membrane is lifted by the tip of the implant (the height shown by the purple line and the portion of the membrane that is under tension shown by the blue line) is less in the narrower maxillary sinus (the red line shows the extent of the stripped mucosa) than in the wider maxillary sinus after membrane stripping.

Figure 4 Illustrations of the modified internal sinus elevation procedure. (A) A Summer bone chisel was used to softly tap the alveolar crest, and this bone block was used as the roof of the area of elevated maxillary sinus floor. (B) Separation of the maxillary sinus floor membrane from the alveolar crest using a mucoperiosteal stripper. (C) Implant placement.

Figure 5 Separation of the maxillary sinus floor membrane/sinus mucoperiosteal detachment procedure.

Figure 6 Surgical procedure for modified internal sinus elevation. (A) Preparation for surgery. (B) The alveolar crest is flat and slightly bluish. (C) Bone block serving as the roof of the area of elevated maxillary sinus floor. (D) Separation of the maxillary sinus floor membrane from the alveolar crest using a mucoperiosteal stripper. (E) Implant placement implant (the bone condensing technique was used to obtain good initial stability). (F) Tight suturing after implantation. (G) CBCT before surgery. (H) CBCT immediately after surgery.

Figure 7 3D reconstruction model operation procedure.

Figure 8 Schematic diagram of the 85° maxillary sinus as an example. Membrane CBCT data were extracted and then fitted to the bone model for matching and fitting.

Figure 9 Reverse processing of solid geometric maxillary sinus bone and membrane models.

Figure 10 Geometric models imported into finite element preprocessing software.

Figure 11 Finite element mesh maxillary sinus and membrane models imported into MSC Patran 2012.

Figure 12 Hyperelastic material parametric equivalent fitting of the maxillary sinus membrane.

Figure 13 Experimental model groupings.

Figure 14 Finite element analysis computational software screen.

Figure 15 Stress distribution nephograms of the sinus membrane.

Figure 16 Peak maxillary sinus floor membrane stress at 0 mm of separation for three working conditions.

Figure 17 Peak maxillary sinus floor membrane stress at 4 mm of separation for three working conditions.

Figure 18 Peak maxillary sinus floor membrane stress at 0 mm and 4 mm of separation for three working conditions.
