# Supplementary material for: Biomechanical analysis of the maxillary sinus floor membrane during internal sinus floor elevation with implants at different angles of the maxillary sinus angles
Source: Int J Implant Dent. 2024 Mar 12;10:11. doi: 10.1186/s40729-024-00530-5 (PMC10933249; doi:10.1186/s40729-024-00530-5)
Supplement: Supplementary file 4 — Supplementary Material 4 [file 40729_2024_530_MOESM4_ESM.docx]

Dear Editor,

Attached, please find our manuscript entitled “Biomechanical analysis of the maxillary sinus floor membrane during internal sinus floor elevation with implants at different angles of the maxillary sinus” by Yinxin Deng *et al.* It is submitted to be considered for publication as an original article in your journal “Clinical Implant Dentistry and Related Research”.

The maxillary sinus elevation has long been considered the most common method for patients with low residual bone height. In this paper, we report a modification of internal sinus elevation by combining the sinus mucoperiosteum stripping procedure and wholly reducing the tension of the sinus mucoperiosteum, which allows for a higher elevation and simultaneous implant placement. Further, we show that the modified internal sinus elevation has a positive clinical effect and demonstrate its rationality in a non-invasive technique by finite element analysis. Thus, this study has revealed, for the first time, that we establish a three-dimensional finite element model of the maxillary sinus membrane（And it is a different angle of the maxillary sinus） and dynamically simulate the modified internal sinus elevation, then study the stress-strain of the maxillary sinus floor membrane. These results may also have implications for promoting and applying the modified internal sinus elevation.

We sincerely hope you also consider this manuscript worthy of consideration for Clinical Implant Dentistry and Related Research. Furthermore, we recommend the following four reviewers to assist in evaluating this paper.

(1) Yi Man

Department of Oral Implantology, West China Hospital of Stomatology, Sichuan University, Chengdu, China

E-mail: [manyi780203@126.com](mailto:manyi780203@126.com)

(2) Dehua Li

Department of Oral Implants, School of Stomatology, State Key Laboratory of Military Stomatology, National Clinical Research Center for Oral Diseases, Shaanxi Engineering Research Center for Dental Materials and Advanced Manufacture, Fourth Military Medical University, Xi’an, Shaanxi, China

E-mail: [lidehua@fmmu.edu.cn](mailto:lidehua@fmmu.edu.cn)

(3) Yucheng Su

Dental Implant Center, Peking Union Medical College Hospital. Beijing, China.

E-mail: [yuchengsu@163.com](mailto:yuchengsu@163.com)

(4) Bernhard Pommer

Department of Oral Surgery, Bernhard Gottlieb School of Dentistry, Medical University of Vienna, Vienna, Austria

E-mail: [bernhard.pommer@meduniwien.ac.at](mailto:bernhard.pommer@meduniwien.ac.at)

I look forward to working with you and the reviewers to move this manuscript closer to publication in the *Clinical Implant Dentistry and Related Research.*

Thank you for your consideration. I look forward to hearing from you.

Sincerely,

Yinxin Deng

Department of Stomatology, Beijing Hospital of Integrated Traditional Chinese and Western Medicine, Beijing, China

e-mail: 1018881917@qq.com
