# Supplementary material for: Biomechanical analysis of the maxillary sinus floor membrane during internal sinus floor elevation with implants at different angles of the maxillary sinus angles
Source: Int J Implant Dent. 2024 Mar 12;10:11. doi: 10.1186/s40729-024-00530-5 (PMC10933249; doi:10.1186/s40729-024-00530-5)
Supplement: Supplementary file 6 — Supplementary Material 6 [file 40729_2024_530_MOESM6_ESM.docx]

**Dear editor and reviewers:**

Thanks very much for taking your time to review this manuscript. I really appreciate all your comments and suggestions! Please check my itemized responses in below and my revisions in the re-submitted manuscript.

After revisions, I have also polished the manuscript's language and would like you to review it again. Thanks again!

**Reviewer #1: How is the sample size calculated?
Are the implants all placed at the same angle? Does it have an effect on the results?**

We sincerely thank you for your careful reading. In the manuscript, we mentioned that we had collected 80 CBCT data of maxillary sinus and measured the angle of these maxillary sinus data; finally, we found that there are five maxillary sinuses <70°, five maxillary sinuses >100°, and the rest of them are all concentrated in the range of 70°- 100°. Although we only measured 80 maxillary sinus data statistically, which is not a very large sample size, it does not affect our final set of three sets of maxillary sinus models with different angles for 3D finite element analysis. Therefore, the 85° maxillary sinus in the median group was chosen to represent a medium-width maxillary sinus (which can also represent the maxillary sinus of most people). A narrower maxillary sinus (45°) and a wider maxillary sinus (125°) were artificially set to obtain a more desirable result with such modeling. The final results of the finite element analysis are also very instructive.

In addition to your question about the placement angle, we would like to clarify that because this manuscript is a biomechanical study based on finite element analysis, we have set the same implantation angle to simulate the modified maxillary sinus elevation. We focus on analyzing the implant tip's effect on the maxillary sinus membrane at different angles. Therefore, the same implantation angle (vertical upward implantation, the most common restorative-oriented implant placement in the clinical situation) was set to simplify the model and reduce possible calculation errors.

Of course, we also know that the actual implantation angle will differ in each case (even if we use guides and other assistive technologies; this error is unavoidable) and that different implantation angles will affect the maxillary sinus membrane differently. Because of this, finite element analysis cannot completely simulate the actual clinical situation, so we have to expand the indications of our modified surgical procedures based on mechanical analysis, and the combination of clinical experiments and finite element mechanical analysis can lay a more solid foundation for our clinical surgery.

Because we found that the difficulty of stripping the mucosa at the base of the sinus and the effectiveness of stripping the sinus varied with different angles of the maxillary sinus during the modified maxillary sinus elevation, we performed statistical measurements of the angle of the maxillary sinus on the CBCT data of the patients who had completed the modified maxillary sinus elevation. The method of measuring the maxillary sinus is also mentioned in the article, and we also followed the method of previous scholars for our maxillary sinus angle measurement and statistics. Measurements were obtained using the self-contained viewing software. The crown of the adjacent tooth and implant neck served as the reference for each measurement. The gap of the augmentation site and implant length were used for calibration. Implant length was used to correct dimensional distortion and magniﬁcation errors (measured implant length divided by actual implant length). All radiographs were measured three times in each direction, and the average value was obtained and analyzed by a trained independent evaluator.

**Reviewer #2: Your study is carefully designed and interesting.
1. I cannot find in the references, that your report about "modified internal sinus elevation facilitates internal sinus elevation".
2. Since no statistical processing was performed in this study, "significantly" in Conclusion should be deleted.**

We sincerely thank you for your valuable suggestions and for endorsing our manuscript, and we apologize for our carelessness. According to your comments, the relevant references about the modified internal maxillary sinus elevation performed by our team have been noted. I have included a citation after the following sentence on Page 3 in the Introduction: “A retrospective clinical study of this modified procedure was performed, and the results^16^ have been successfully published in the Journal of Clinical Implant Dentistry and Related Research.”; the inappropriate use of the word “significantly” in the conclusion part of the manuscript has also been changed in de conclusion: “In summary, the risk of maxillary sinus floor membrane rupture is greatly reduced after adequate stripping of the sinus floor membrane when performing modified internal sinus elevation in a narrow maxillary sinus.”

Both of these two revisions I have highlighted in the manuscript with a yellow background, please review it again, thank you!
